# Dating the Noceto Vasca Votiva, a unique wooden structure of the 15th century BCE, and the timing of a major societal change in the Bronze Age of northern Italy

**DOI:** 10.1371/journal.pone.0251341

**Published:** 2021-06-09

**Authors:** Mauro Cremaschi, Carol Griggs, Cynthia Kocik, Angela Mutti, Andrea Zerboni, Sturt W. Manning

**Affiliations:** 1 Dipartimento di Scienze della Terra "A. Desio", Università degli Studi di Milano, Milano, Italy; 2 Cornell Tree-Ring Laboratory, Department of Classics, B48 Goldwin Smith Hall, Cornell University, Ithaca, NY, United States of America; 3 Mississippi Valley Archaeology Center, University of Wisconsin-La Crosse, La Crosse, WI, United States of America; 4 Complesso Monumentale della Pilotta–Museo Archeologico, Ministero della Cultura, Parma, Italy; 5 The Cyprus Institute, Nicosia, Cyprus; University at Buffalo - The State University of New York, UNITED STATES

## Abstract

The Noceto ‘Vasca Votiva’ (votive tank), discovered in excavations on a terrace at the southern edge of the Po Plain, northern Italy, is a unique well-preserved wooden (primarily oak) structure dated to the advanced through late Middle Bronze Age (~1600–1300 BCE). This complex monument, comprising two super-imposed tanks, is generally linked with an important but uncertain ritual role involving water. The context provides extraordinary preservation of both wooden, other organic, and cultural finds. The key question until now, hindering further interpretation of this remarkable structure, has been the precise date of the tanks. Initial work pointed to use of the two tanks over about a century. Using dendrochronology and radiocarbon ‘wiggle-matching’ we report near-absolute construction dates for both of the tanks. The lower (older) tank is dated ~1444±4 BCE and the upper (more recent) tank is dated 12 years later at ~1432±4 BCE. This dating of the construction of the Noceto tanks in the 3^rd^ quarter of the 15^th^ century BCE further enables us to reassess the overall period of activity of this wooden complex and its association with a major period of societal change in the Bronze Age of northern Italy starting in the later 15^th^ century BCE.

## Introduction

Recent archaeological excavations uncovered on top of a terrace at the southern edge of the Po Plain of northern Italy a remarkable wooden structure that is unique in world prehistory. The Noceto ‘Vasca Votiva’ (votive tank), is a large wooden plank-lined rectangular tank measuring 12 x 7m (http://www.comune.noceto.pr.it/la-vasca-votiva-di-noceto), and, according to the associated material culture and its standard chronological assessment [1], is dated to the advanced Middle Bronze Age 2 (BM2B) and Middle Bronze Age 3 (BM3) with use perhaps extending towards the beginning of the Recent Bronze Age (within overall limits ~1600–1300 BCE) [2, 3]. Note: in Italian prehistory the later part of the Bronze Age is divided into a ‘Bronzo recente’ and then a ‘Bronzo finale’—the Noceto context may run into the former, and hence we use the term ‘Recent Bronze Age’ from here onwards instead of Late Bronze Age. The wooden structure–primarily built of oak wood (*Quercus* sp.)–was set into the ground, likely for ritual purposes [2]. The monument is complex. It consists of two superimposed wooden tanks. The uppermost (Vasca Superiore or Upper Tank), and better preserved, tank was the second (later) structure at the location. Beneath this were the partial remains of an older wooden structure (Vasca Inferiore or Lower Tank), that had collapsed during or after building. The infilling deposits from the structures exhibit features indicating sedimentary infilling related to sedimentation in water, with alternating thinly laminated fine-grained strata formed after decantation, with peaty layers related to the accumulation of organics, and then occasionally colluvial layers at the margins of the structure. The deposit formed during the life of the local Bronze Age settlement and includes a variety of well-preserved archaeological materials.

The fully buried context created an excellent–anoxic–environment for the preservation of the organic material. As a result, the majority of the timber structure remains were retrieved, providing an extensive archive of evidence for prehistoric wood-working and construction. The sedimentary infilling of the tank included a range of wooden tools and a rich assemblage of ceramic vessels that were intentionally deposited in association with the tank–the latter material and apparent repeated depositional behaviours suggest ritual activities, and thus a primarily ritual function for the tank.

The obvious outstanding question after the initial work on the tank and its assemblage was determining a more precise date for this unique structure. Preliminary work indicated that the two structures were in use for about a century ~1420–1320 BCE [2, 4]. We report here on a new dating investigation of the Noceto ‘Vasca Votiva’ comprising a combination of dendrochronology and radiocarbon (^14^C) ‘wiggle-match’ dating to provide (i) a near-absolute construction date for the Vasca Superiore in the 3^rd^ quarter of the 15^th^ century BCE and (ii) to allow a reassessment of the overall period of activity associated with the construction of this unique wooden complex.

### Archaeological context

**Fig 1:**
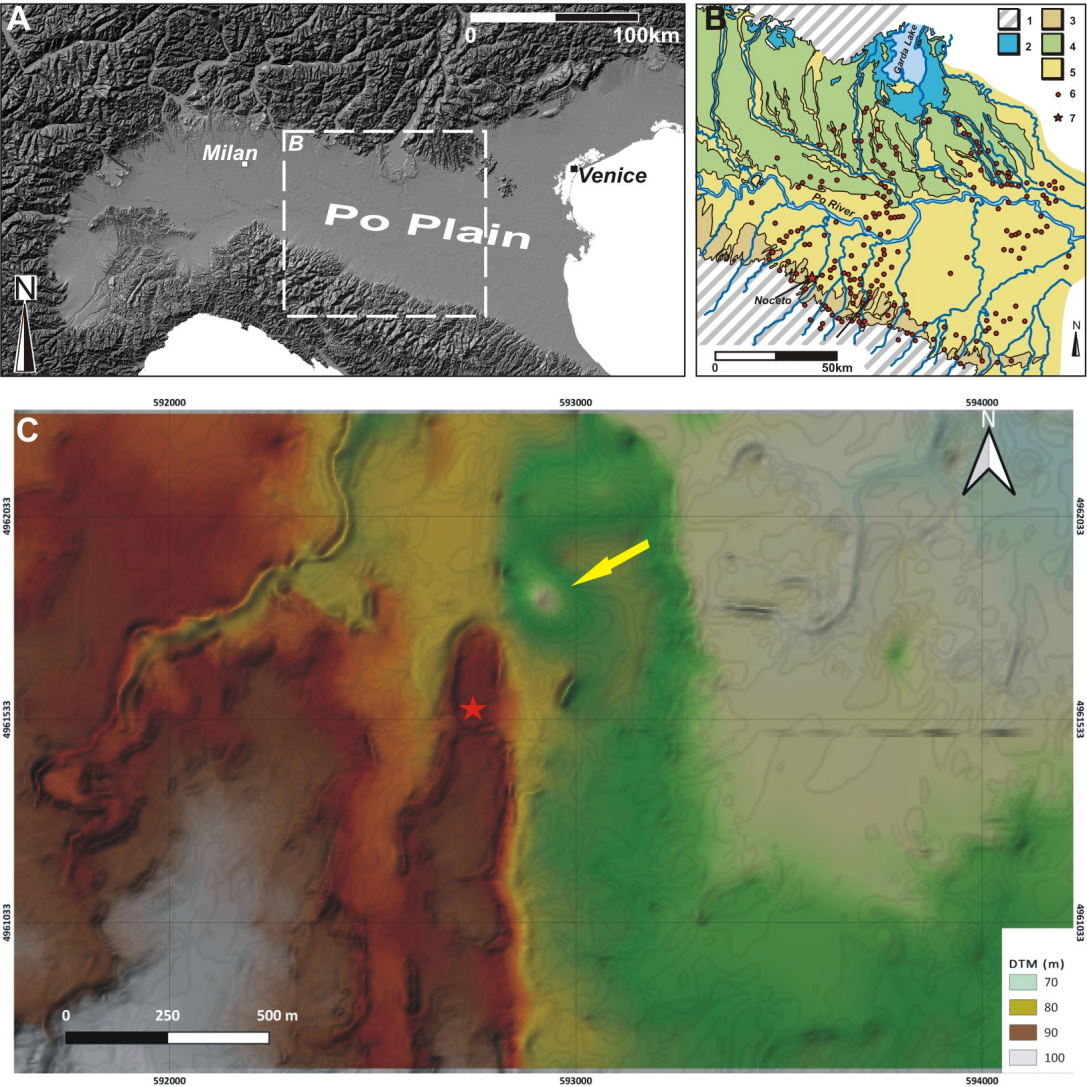
The location of the Noceto site. (A) Digital terrain model of northern Italy illustrating the areal distribution of the Terramare culture along the Po Plain. (B) Simplified geomorphological sketch of the region illustrating the distribution of the Terramare settlements (dots) and the position of the Noceto settlement (star) (modified from [5]). (C) DTM of the Noceto area illustrating the position of the Vasca Votiva (star) and the depression related to the quarry (arrow), which was the location of the Bronze Age settlement. Key for (B): 1, pre-Quaternary formations; 2, Alpine Pleistocene glacial deposits; 3, Pleistocene deposits at the foot of the Apennine; 4, Pre-Holocene terraces; 5, alluvial plain.

**Fig 2:**
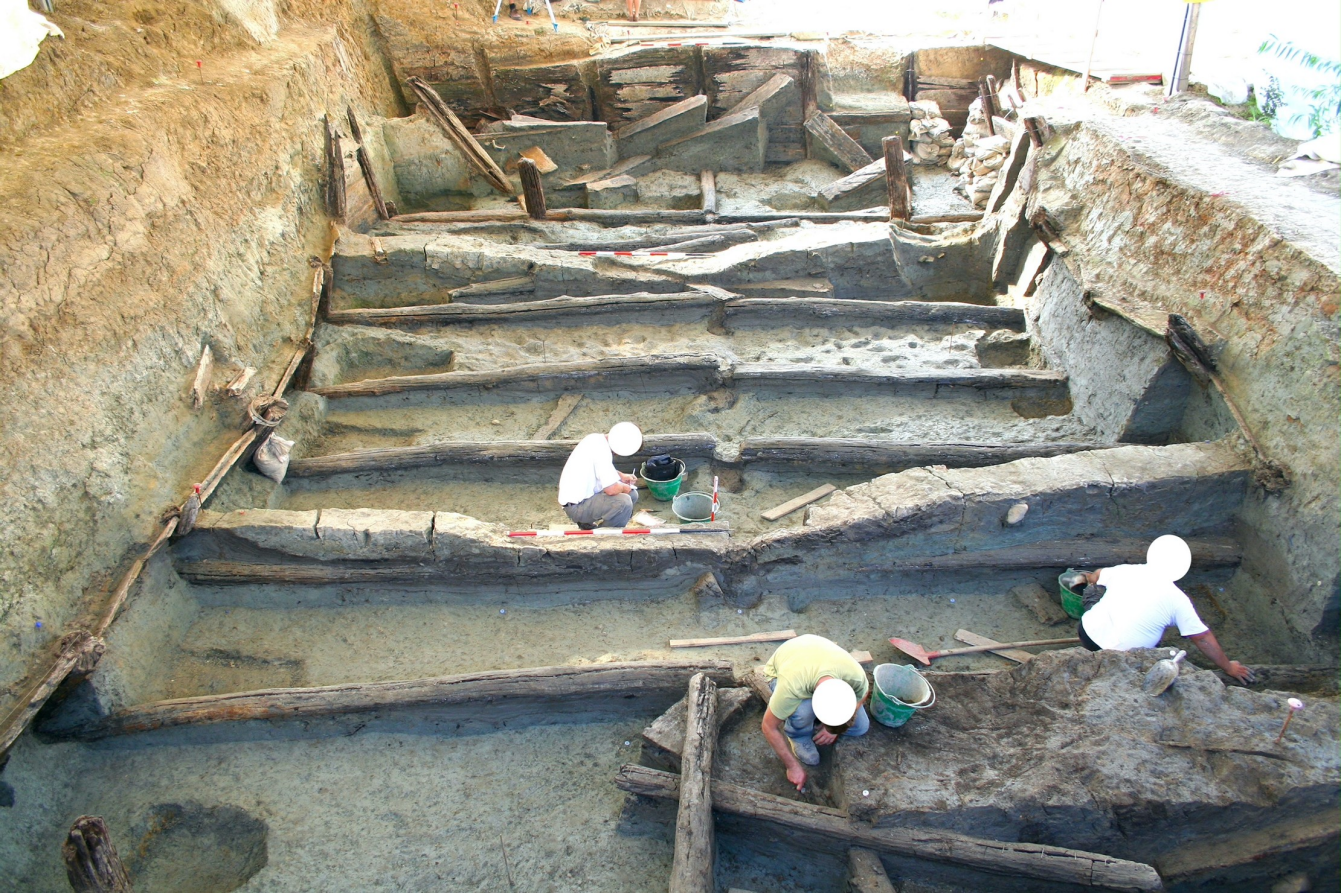
The lower tank at Noceto under excavation.

**Fig 3:**
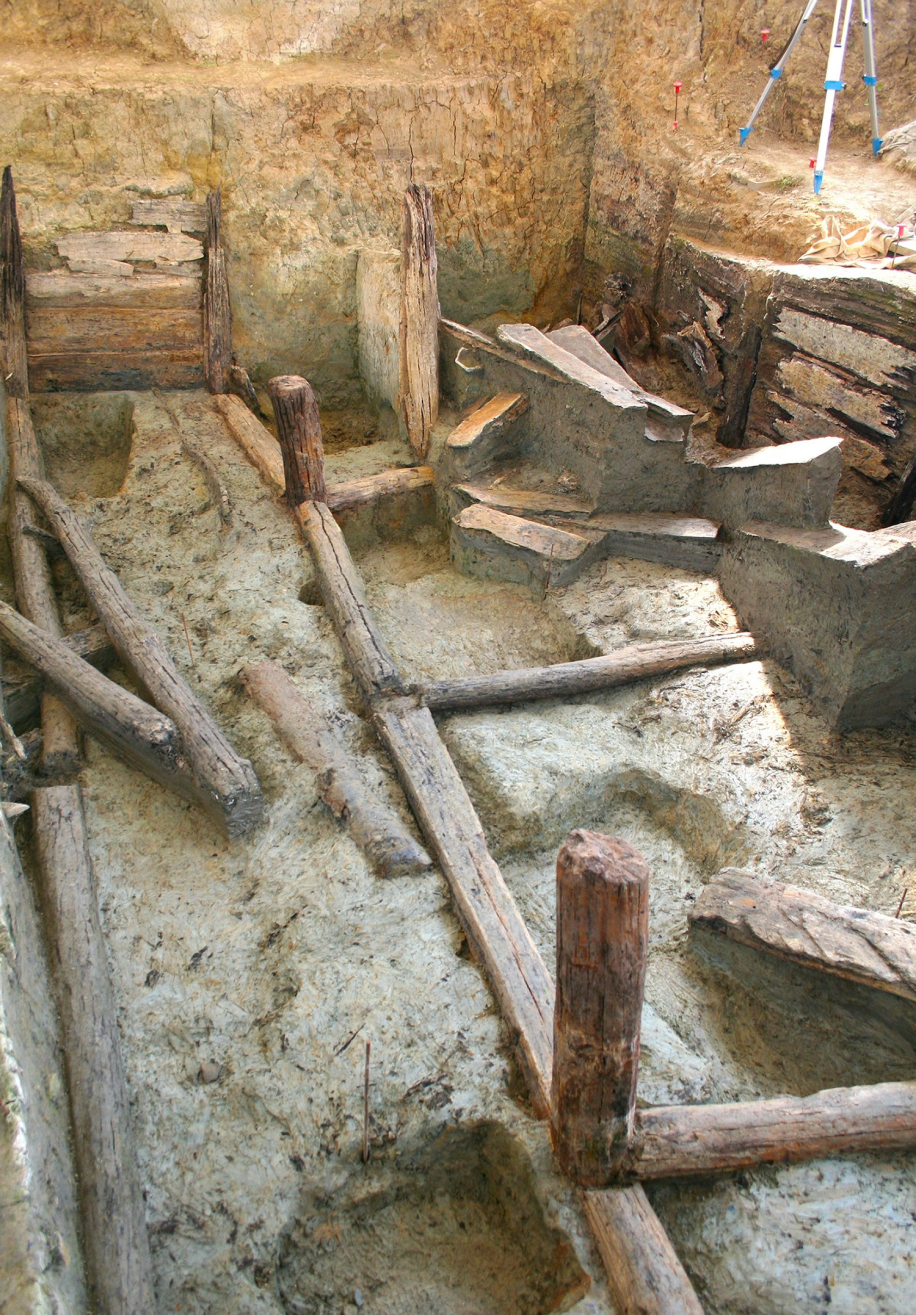
Details of the basal network of beams from the lower tank at Noceto.

The Noceto Vasca Votiva came to light during construction works, at the periphery of Noceto (Parma, Northern Italy), near a Terramare (Middle-Recent Bronze Age culture) settlement that had been largely destroyed by quarrying in the 19th century CE [2] (Fig 1). The structure, made up of wood poles, beams and boards, covered and strengthened the walls of a deep pit excavated into the geological substrate (clay) of a terrace, and was aimed at containing water: it thus formed an artificial basin, in which votive offerings were deposited several times. It is composed of two wooden structures, which while found penetrating each other, may be distinguished because they were constructed using different techniques. The lower tank (Fig 2) reaches a depth of four meters from the extant ground level. It is composed of 36 vertical poles, fixed into the substrate at regular intervals that delimit a rectangular perimeter of 9 x 19m. The poles have a longitudinal groove on two opposite sides in which horizontal elements are locked to form a continuous curtain, arranged to support the walls of the pit. Posts and boards are held at their bottom by a complex network of horizontal beams and anchored to a further row of posts driven into the center of the pit (Fig 3). The lower (older) tank was never finished. Numerous clues (shavings linked to the woodworking in place, abandoned construction tools, parts of the structure removed) indicate that profound modifications of the structure were underway when its walls yielded to the thrust of the clays in which they were dug and collapsed. The debris resulting from the collapse were partly removed and inside the same pit, the upper tank was then built. Some of the wood items from the pre-existing tank were reused to build the new structure, but the new architectural plan was substantially different and aimed to guarantee that the structure would be able successfully to withstand the pressure from the walls.

**Fig 4:**
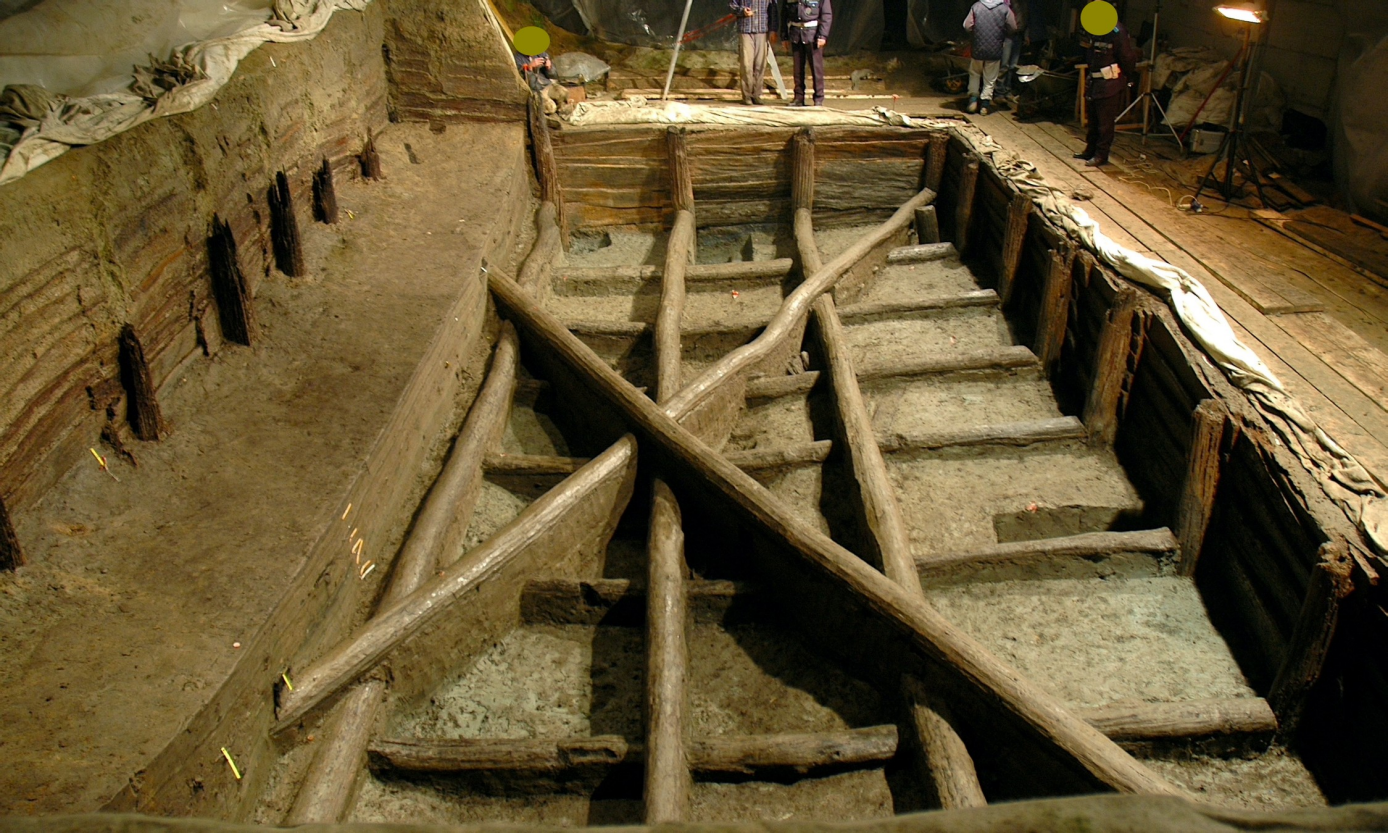
The upper tank at Noceto during the excavation.

**Fig 5:**
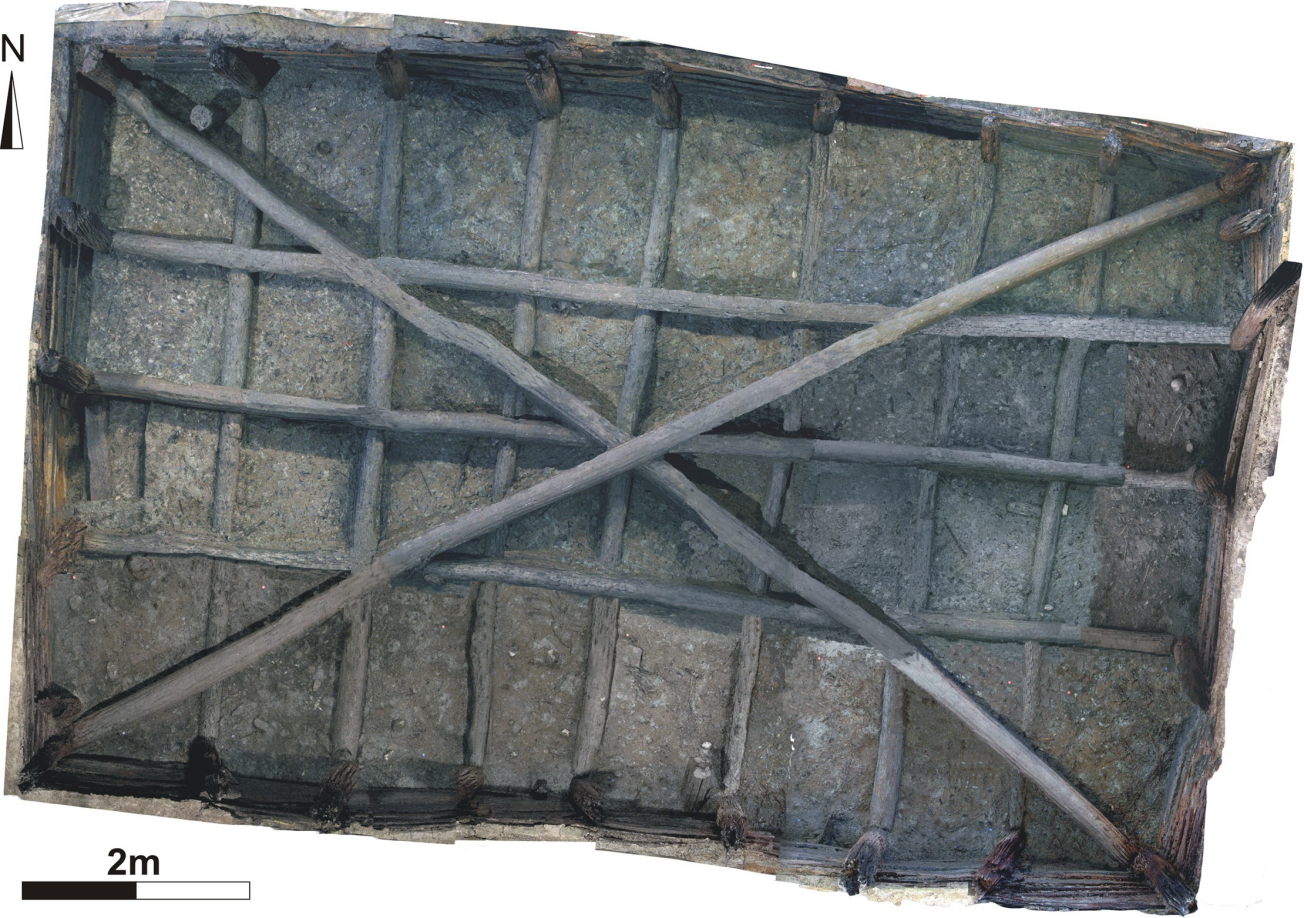
Overhead photogrammetric plan image of the upper tank at Noceto.

**Fig 6:**
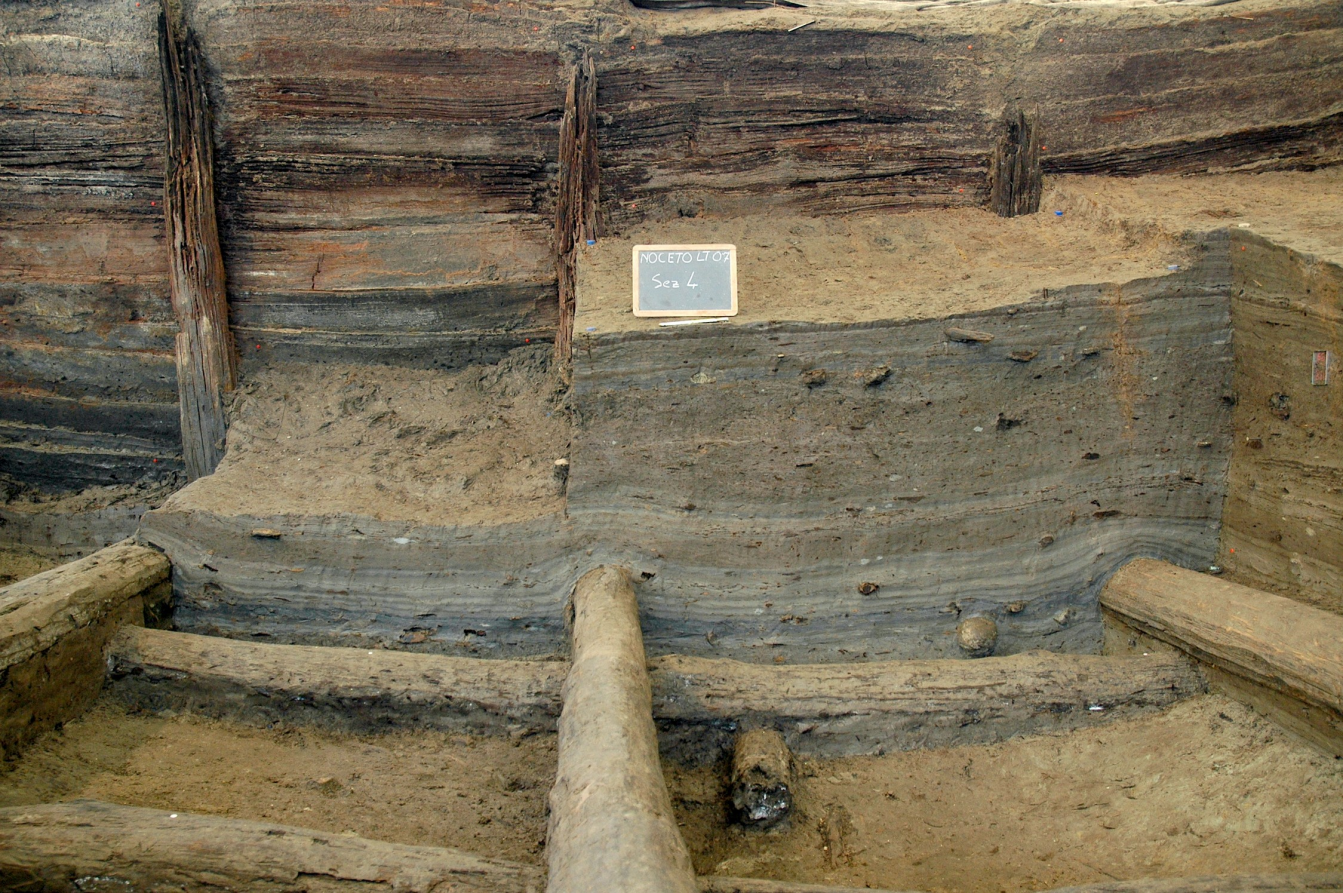
The fill of the upper tank at Noceto showing the laminated deposits lying upon the beams placed at the base of the structure.

The upper tank, while maintaining a width close to the previous one, is significantly shorter. It consists of 26 poles arranged vertically at regular intervals along a rectangular perimeter of about 7 x 12m (Fig 4). The poles hold the horizontal elements (over 240) that line the walls, partly overlapping one another (both vertically and horizontally) pressed against the earthen walls of the pit in order to form a solid and coherent (inter-locked) structure. In turn, the poles are blocked, at the bottom and at the top, by two networks of beams arranged horizontally perpendicular to each other to which are added two long beams (up to 13m long) crossing the tank and supporting the four poles placed at each corner of the tank. The different wooden elements were all carefully polished with axes and, since no shavings were found at the base of the filling of the upper tank, it can be deduced that they must have been transported to the site already finished (Fig 5). The sedimentary infilling is made up of silts, sandy silts and peaty silts and gyttja, organized as thin layers, with direct gradation, sometimes interspersed with beds of diatoms [6] (Fig 6). Such a deposition record testifies that the final, in-use, structure was filled with water. It is likely that the high impermeability of the clays of the surrounding bedrock helped to contain the water in a stationary, still, pool with sediment slowly settling at the base of the pool (decantation). The exceptional preservation of organics and the occurrence of extensive mineralization of vivianite in the sediments, and attached to the wood items, suggest that sedimentation took place under anoxic conditions. The same environmental settings allowed the near perfect preservation of the complex architectural structure.

**Fig 7:**
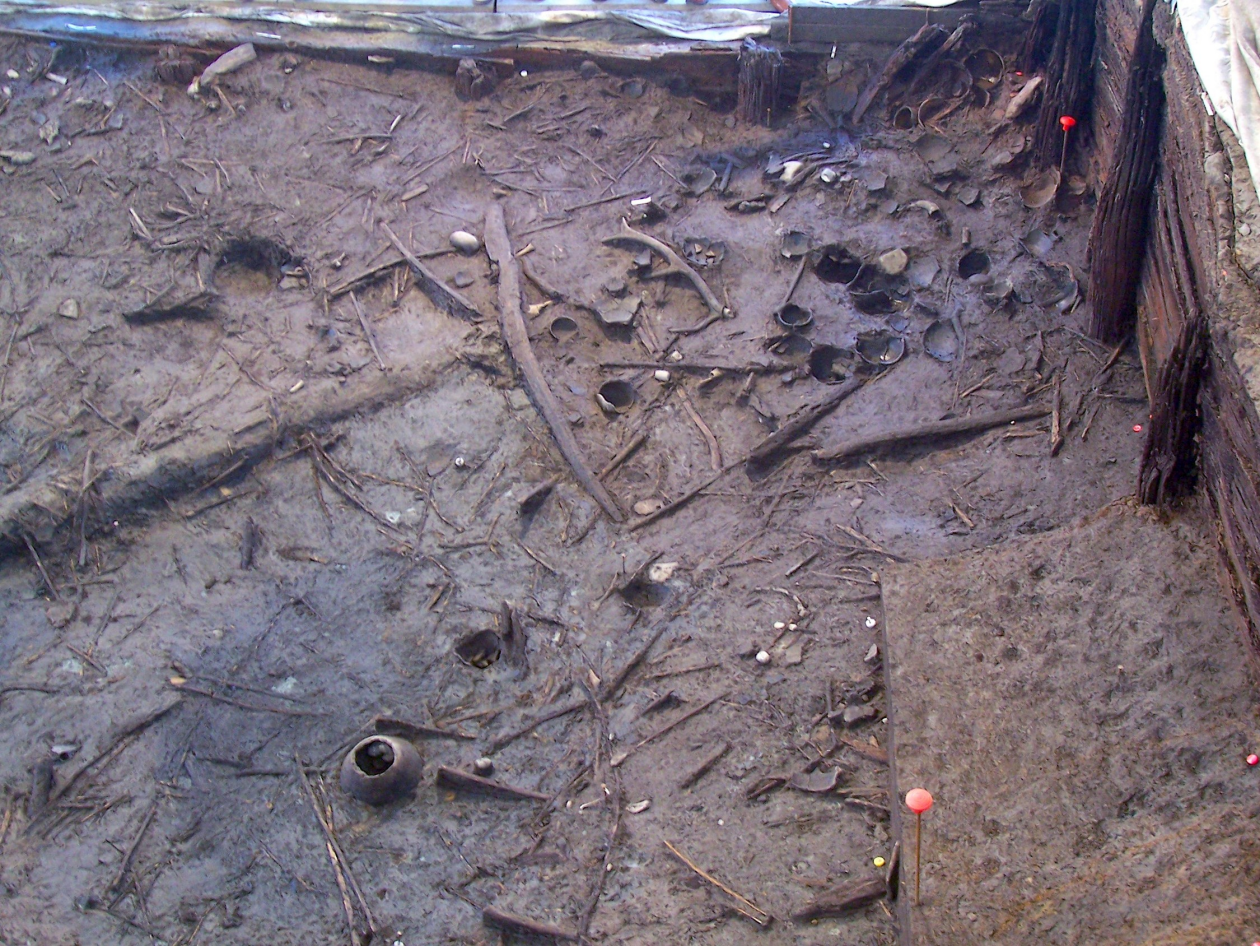
Some of the Bronze Age vessels and wooden items, deposited in the upper tank at Noceto (in situ).

The fill deposited in the upper tank includes an extraordinary set of archaeological items (stone, wood and ceramic), which were placed voluntarily, in at least three separate episodes during the lifetime of the upper structure (Fig 7). Archaeological items were lowered to the bottom of the tank full of water and then slowly buried by the progress of sedimentation. These depositions are presumably ritual (some form of water cult?) and include about 150 whole vases, 25 miniature vessels, 7 clay figurines, numerous examples of baskets, handles or bands, spindles, shovels and four plows or parts of these made of wood or other vegetal tissue [2]. Additionally, we found exotic pebbles, woods deriving from harvest in forest areas, and faunal remains [7, 8]. In the lower tank, there is further evidence of at least one deposition [9], thus indicating that even for a limited period this tank was also in use before its collapse (and the subsequent rebuilding comprising the upper tank).

**Fig 8:**
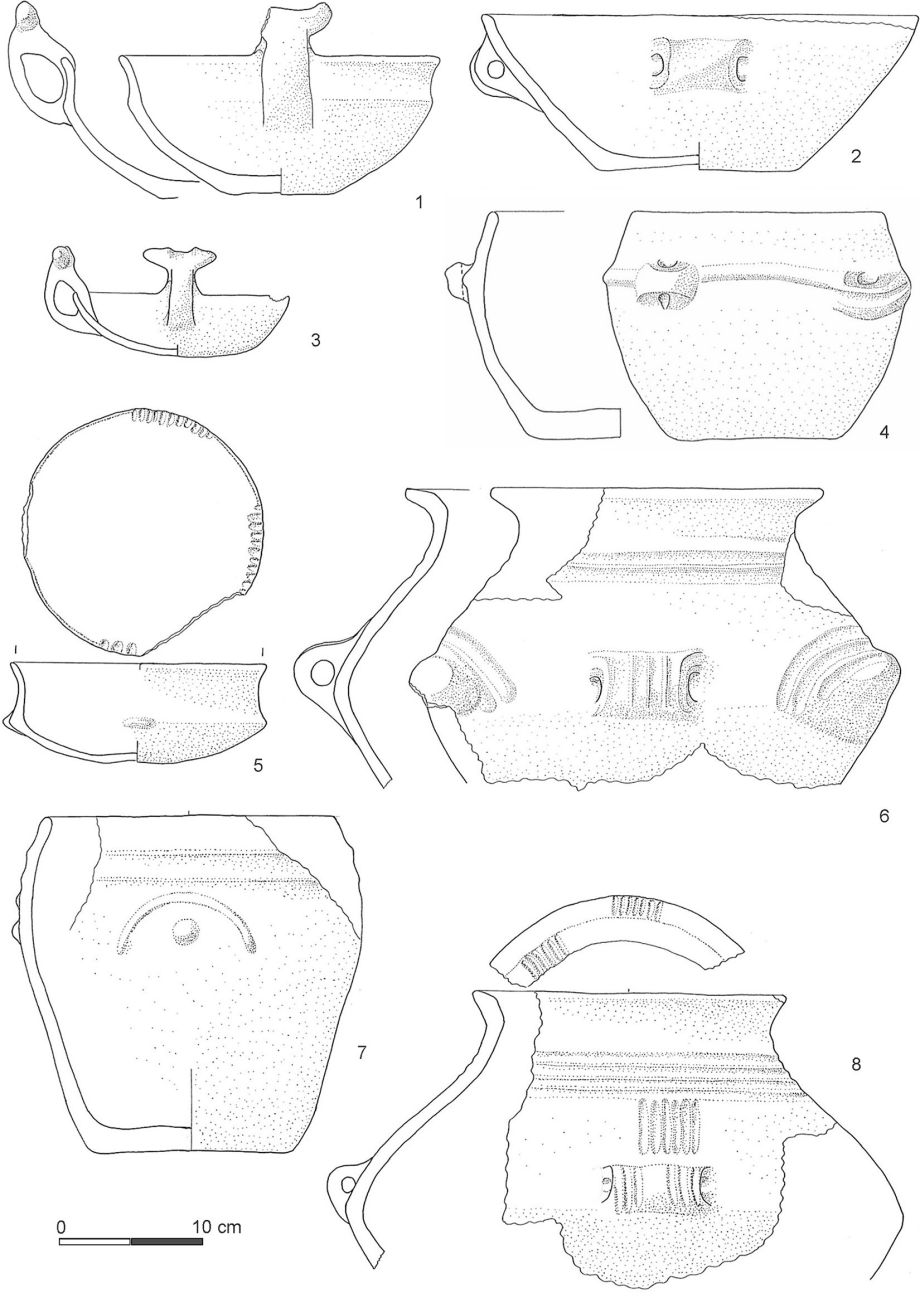
Selection of archaeological material from the lower tank dating to the BM2B phase (from [3, 9]). Pots 1, 3, 4, 5 and 7 are from SU 172 alfa (this SU is associated to the ^14^C age UGAMS-29349 in Table 4 below); pots 2, 6, 8 are from SU 542.

**Fig 9:**
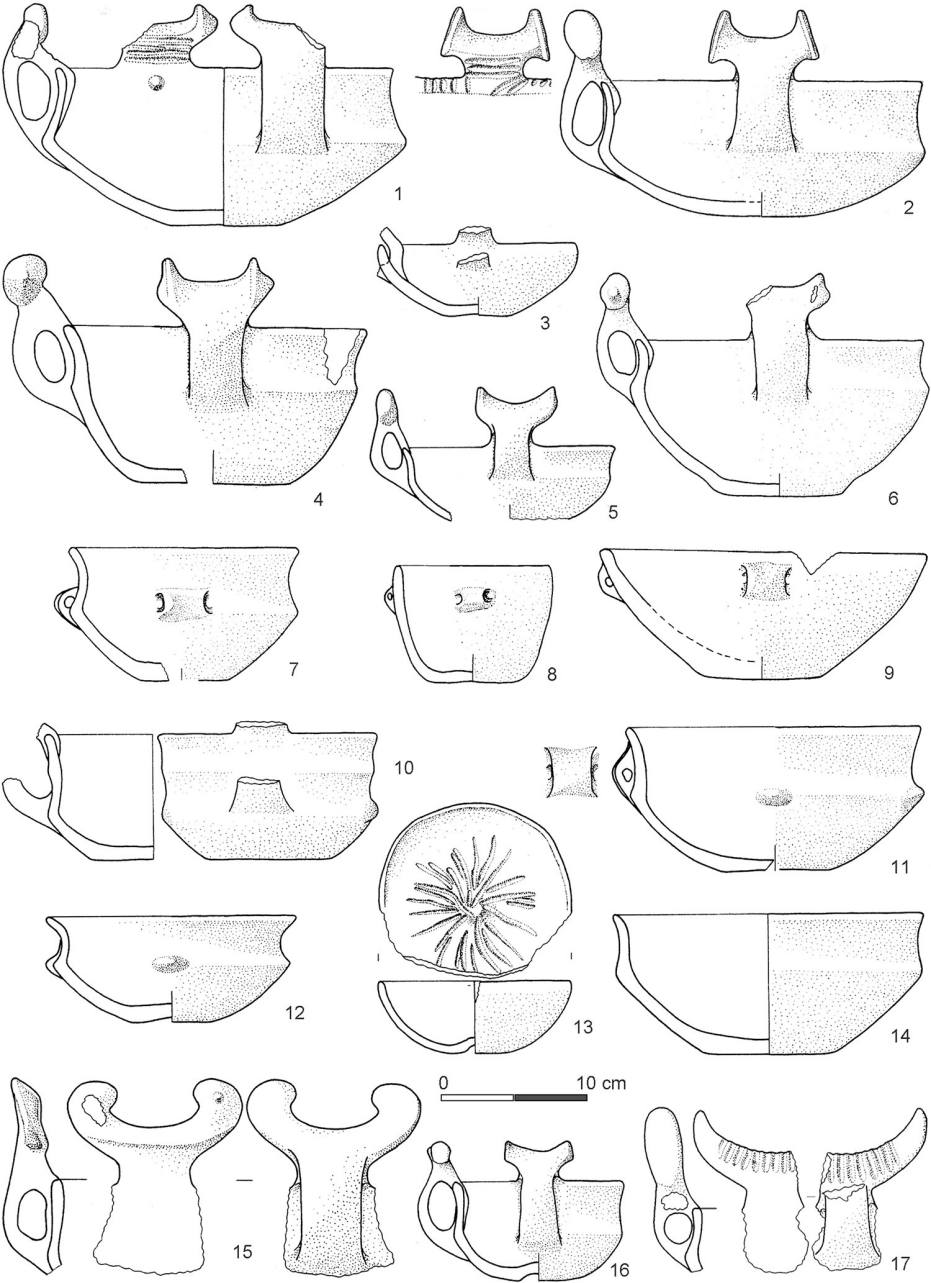
Selection of archaeological material from the upper tank dating to the BM3 phase (from [10]). Pots 1 and 2 are from SU 87a; pots 3 and 9 are from SUs 81, 81c, 81d-e; pots 10 and 14 are from SU 66base; pot 15 is from SU 5003 (this SU is associated to the ^14^C age Poz-19307 in Table 4 below); pots 16 and 17 are from SU 5002c-e.

From a chronological-typological assessment, the pottery vessels contained in the tank illustrate a significant chronological gradient between the bottom and the top of the stratigraphic sequence [3, 9–11]. Pottery found in the infilling of the lower tank dates to a specific phase of the later Middle Bronze Age 2 period, BM2B (from the second half of the 16th century BCE to the middle of the 15th century BCE, or ~1550–1450 BCE) (Fig 8), whereas the material from the upper tank belongs to a later period, Middle Bronze Age 3, BM3, placed between the beginning of the 15th century BCE and the end of the 14th century BCE or ~1400–1350/00 BCE [1, 8–11] (Fig 9). It is therefore evident that conventional archaeological dating has only a low resolution and suggests a wide time-window for the building and use of the monument, covering anywhere from ~50 to ~250 years. This time-window is too large and too unsatisfactorily resolved to enable progress towards any detailed questions about the timing of the construction of the tanks, how long they were used for, and therefore investigations into topics of how, why and by whom. In particular, to engage in discussion of the meaning and use of the tanks—their biography—it is key to understand exactly (i) when they were built and whether each tank was a single construction episode, (ii) how much time elapsed between the construction of the lower tank and then its collapse before (iii) the building of the upper tank, and (iv) how long the upper tank subsequently remained in use as an active monumental entity.

Here we address these issues thanks to the concomitant presence at Noceto of three dating sources: (1) well preserved and typologically significant archaeological material, (2) the presence of short-lived organic material for ^14^C dating, and (3) wooden elements obtained from mature oak (*Quercus* sp.) trees, a number with long tree-ring sequences, that are therefore suitable for dendrochronological analysis.

## Materials and methods

### Materials

All specimens investigated in this study were sampled with the authorization of the Ministero dei Beni e delle Attività Culturali e del Turismo (MiBACT) of Italy (now Ministero della Cultura–MiC); the same ministry, through Soprintendenza ai Beni Archeologici dell’ Emilia Romagna, sponsored and enabled the excavation which was carried out in cooperation with the University of Milan. Archaeological materials are stored at the Museo Archeologico della Vasca Votiva in the municipality of Noceto (PR, northern Italy). A total of 28 tree-ring samples from the buried wooden structure at the Noceto site were examined at the Cornell Tree-Ring Laboratory (CTRL) from December 2016 through earlier 2020. Of the 28 samples, NOC-1 to 28, 25 are of oak and 3 of elm species (*Quercus* sp. and *Ulmus* sp., respectively) (Tables 1 and 2). The building and structural timbers are very well-preserved despite the >3000 years since felling, construction, use, and abandonment. Twenty of the 25 oak samples are from boards made of wedge-shaped radial sections of less than an eighth of a complete cross section, and many of the boards came from the same tree. The other eight samples, five oak and three elm, are half to full cross-sections from whole logs. Five, and possibly another two, of the oak samples had been treated for preservation, likely with polyethylene glycol (PEG), prior to being sent to Cornell. While the PEG did not impede dendrochronological analysis, the treated samples were not used for ^14^C dating.

**Table 1:** Find locations of the samples from the two superposed Noceto tanks and the relation with the two phases identified by the dendrochronological analysis.

Noceto Element ID and Structure Association	CTRL ID	Noceto Phase	Noceto Location	Description of wooden element
***UPPER STRUCTURE***				
N15	NOC 1	(Elm)	D4	post
N170	NOC 2	PH 1	D4	board/plank
N 173	NOC 3	PH 1	D14	board/plank
N 174	NOC 4	PH 1	D 13	board/plank
N 3	NOC 22	PH2	D13	board/plank
N 169 (palo SE)	NOC 23	PH 1	L 15	post
N 152	NOC 27	PH 2	I 13	beam
N 151	NOC 26	PH 2	G 13	beam
N 158	NOC 28	PH2	F 13	beam
***LOWER STRUCTURE***				
N 548	NOC 5	PH1	D 16–17	board/plank
N 581	NOC 7	PH1	H-I 17	board/plank
N 582	NOC 8	PH1	H-I 17	board/plank
N 599	NOC 9	PH1	H-I 17	board/plank
N 601	NOC 10	PH 1	L-M 17	board/plank
N 603	NOC 21	PH 1	L-M 17	board/plank
N 550	NOC 6	PH 1	E 16–17	board/plank
N 569	NOC 24	(Elm)	DE 17	beam/pole
N 485 (transversal pole)	NOC 13	(Elm)	G 14	beam
N 599 (vertical pole NE)	NOC 25	PH 1	D 17	post
N 547	NOC 11	PH2	DE 16–17	board/plank
N 561	NOC 12	PH1	E 14–15	board/plank
N 573	NOC 14	PH 1	E 17	board/plank
N 574	NOC 15	PH 1	E 17	board/plank
N 582 bis	NOC 16	PH 1	H-I 17	board/plank
N 585	NOC 17	PH1	I-L 17	board/plank
N 589	NOC 18	PH1	C-D-E 17	board/plank
N 606	NOC 20	PH1	H-I 18	board/plank
N 715	NOC 19	PH 1	I-L 18	board/plank

**Table 2:** Descriptions of the 14 trees represented by the 25 oak samples and the 2 elm trees represented by 3 samples.

CTRL Lab Sample Numbers	Site Sample Names and Numbers	Max. dim, cm	R or D	Board or Section	N*	RY beg	RY ends	SW count	Outer ring
**Oak samples**									
***Phase 1***									
NOC-3, 4, & 18	N173, N174, N 589	26.0	R	B	207	1000	1206	23	v
NOC-14, 19, & 21	N 573, N 715, N 603	32.3	R	B	225	981	1205	19	v
NOC-6, 8, 12, 15, 16, & 20	N550, N582, N 561, N 574, N 582 bis, N 606	29.0	R	B	190	1016	1205	19	v
NOC-25	Palo Verticale NE	12.1	R	S	105	1100	1204	9	v
NOC-5 & 17	N548, N 585	25.5	R	B	198	1007	1204	13	v
NOC-7	N581	24.5	R	S	202	1003	1204	14	v
NOC-2 & 9	N170, N599	25.0	R	B	167	1033	1199	7	v
NOC-10	N601	23.7	R	S	200	1000	1199	27	v
NOC-23	Palo SE Vasca Superiore	9.5	R	S	73	1114	1186	3	vv
***Phase 2***									
NOC-27	US152	30.0	D	S	108	1111	1218	10	W
NOC-22	US3	30.8	R	B	281	938	1218	18	v
NOC-26	US151	15.0	D	S	99	1118	1216	7	v
NOC-11	N 547	29.0	R	S	181	1021	1201	1	vv
NOC-28	US158	15.0	D	S	71	1129	1199	0	vv
**Elm Samples**									
NOC-1	N15	5.7	R	S	27	NA	NA	0	vv
NOC-24&13	N 569, Palo Trasversale	19.7	D	S	74	NA	NA	27	v

CTRL = Cornell Tree-Ring Laboratory sample ID, ‘Max dim’ = maximum dimension on transverse surface in cm, R or D = Radius or Diameter; N* = ring count, including partial and unmeasured complete rings; RY beg = relative years begin; RY end = relative years end; SW count = sapwood ring count; Outer ring: W = waney edge; v = close to waney edge; vv = unknown number of rings missing between outer ring and waney edge.

### Find locations of the samples

**Fig 10:**
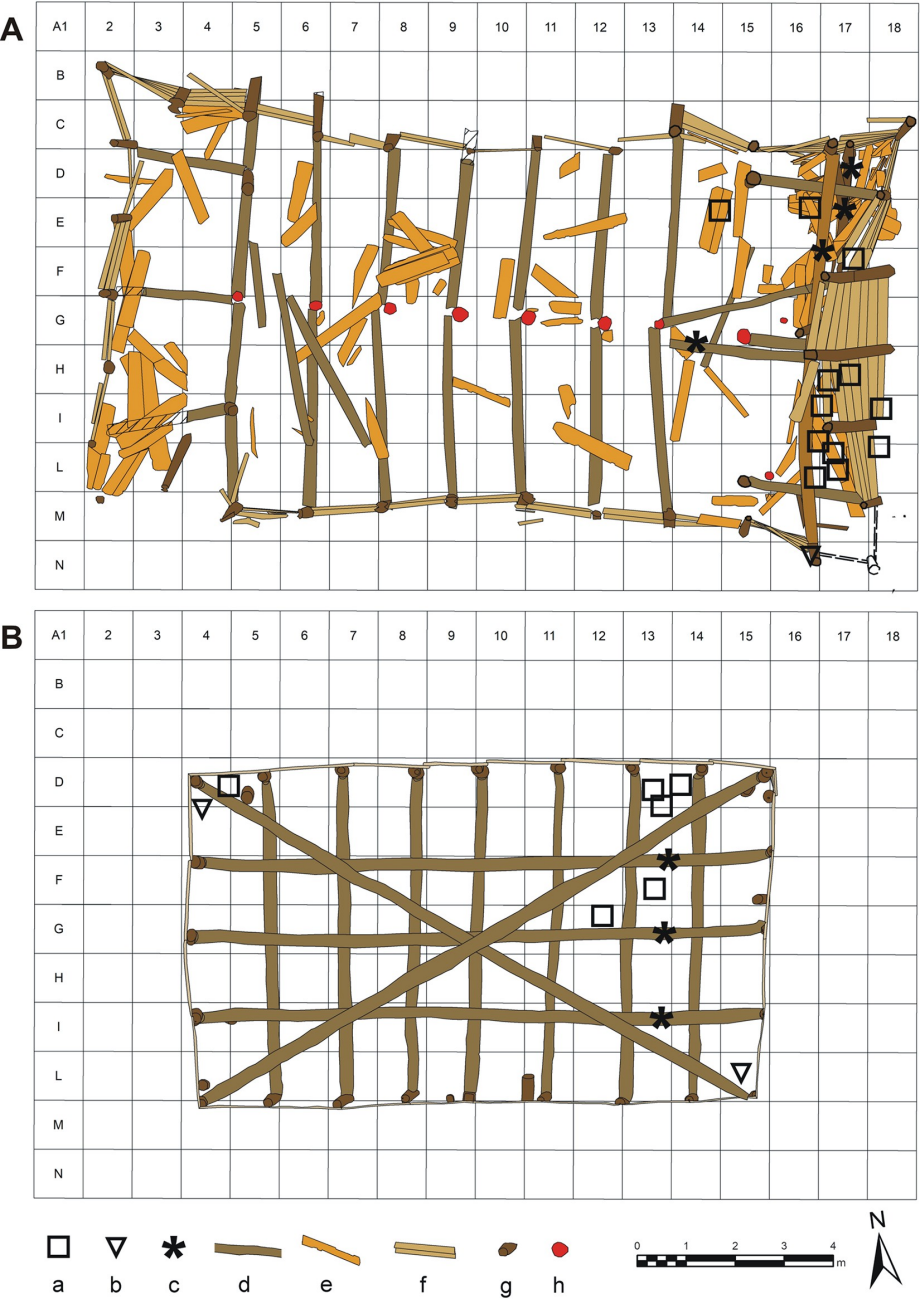
Location of the samples submitted to the dendrochronological analysis. (A) lower structure, (B) upper structure: a) sample of board, b) sample of post, c) sample of beam, d) beam, e) scattered board inside the lower structure, f) standing board, g) post, h) post hole.

Of the 28 samples analyzed, 9 come from the upper tank (2 poles, 3 beams, 4 horizontal elements = boards/planks) (Fig 10). The 3 beams belong securely to the original construction of the upper tank base structure. The 4 horizontal elements (boards/planks) and 2 poles come from the fill of the tank and represent elements from the upper part of the wooden structures that have subsequently (post-use) fallen/collapsed into the tank fill. The remaining 19 samples come from the lower tank. These consist of 16 horizontal elements (boards/planks), all coming from the eastern wall of the structure overturned in the tank due to the collapse of the wall. Moreover, we considered also one sample of the vertical pole of the NE corner, a horizontal beam of the basal structure, and the pole/beam used to reinforce the opposing walls of the tank.

### Tree sample preparation

The samples were rinsed, and those treated with PEG were also soaked in warm water for approximately two hours to dissolve some of the compound. Dimensions were recorded and an initial assessment and recording made of each sample, including ring count, potential for successful ring measurement, presence or absence of sapwood and waney edge (the terminal growth increment/tree-ring immediately under the bark), and identification to species by comparison of physiological features with standard European-Mediterranean tree wood reference materials and sources [12–14] and the InsideWood online database (https://insidewood.lib.ncsu.edu/). On the transverse surface of each sample, at least two radii were chosen for ring-width measurements. The radii were prepared by using razor blades to expose ring boundaries. Water was used to soften the surface for preparation, to enhance the view of ring boundaries during measurement, and to avoid dehydration. The samples were then kept wrapped in plastic and stored in sealed bags. Any samples received with mold were treated with a 5% bleach or 70% isopropyl alcohol solution to remove mold and inhibit any further growth.

Ring-widths were measured at 0.01 mm precision along the prepared radii from the innermost complete ring to the outermost complete and measurable ring using a traveling stage under a stereo microscope with crosshairs using the Tellervo software package [15, 16]. Incomplete rings, and/or distorted rings from the presence of irregular ring growth, drying, or other damage, were counted but not measured. Readings of radii from the same sample were averaged into a single ring-width sequence, but if the two radial sequences were significantly different, the radius in which rings had the least idiosyncratic growth (e.g. the radius least affected by growth scars or reaction cells) was selected for analysis. The sequences of different samples were compared to each other to identify samples from the same tree, and those judged to be from the same tree were averaged together. The sequence for each tree was then detrended by using standard dendrochronological methods to remove the variations in ring widths unique to each tree to preserve the “common signal” that is present in all the represented trees (e.g. [17]). The growth patterns in the time series of each tree were then compared with one another, both visually, and statistically, to find matching growth patterns, and thus to relatively place (crossdate) them where possible one to another in time following standard dendrochronological principles and practices (e.g. [17–20]). Statistics employed to determine the degree of similarity between sequences include the modified version of Student’s t-score *t* (after [21]), Pearson’s correlation coefficient *r*, and the trend coefficient *tr* (after [22] modified by [23]) using the implementations in Tellervo [15, 16]. The trend coefficient is determined by the number of times that both sequences increase or decrease from year-to-year during years of overlap. Relatively-dated samples were combined into chronologies, and their outer rings examined to find those with possible waney edges in order to identify evidence of, and so relative dates of, possible building phases, modifications, or repair among the collected samples. Specific tree-rings selected for ^14^C dating were dissected using a steel blade under a binocular microscope. The NOC tree-ring measurement data are in the S1 File.

### Radiocarbon dates and analysis

Seventeen ^14^C dates were obtained on 11 tree-ring samples, processed either to α-cellulose or holocellulose (see Table 3), from the Centre for Isotope Research (CIO) at Groningen using published methods [24]. One of the ^14^C measurements, GrM-11275, is regarded as an unexplained large outlier (too old), and this sample was re-measured, as GrM-17406, and this date only is used in our analyses (and GrM-11275 is not used in this study). Calibration into calendar years and ^14^C modelling employed the OxCal software [26–28] using version 4.4.2 with the IntCal20 Northern Hemisphere ^14^C calibration curve [29] (with curve resolution set at 1 year–we also tested using a curve resolution of 5 years) and otherwise the default program settings. Where ^14^C dates comprised the same (cross-dated) tree-rings or mid-points, and so represent estimates of the same ^14^C date/calendar age relationship, we combined these into weighted averages [30] using the R_Combine function in OxCal. The sets of tree-rings comprising each sample were analyzed as best representing the date of the mid-point of the set (e.g. for Relative Years, RY, 1 to 5 this would be RY3). A test for individual outliers used the OxCal SSimple Outlier model [27]. The SSimple Outlier model was also used to assess weighted averages against the model. The calendar placement of the tree-ring-sequenced ^14^C time series employed ‘wiggle-matching’ [28, 31] using two methods: (i) the D_Sequence function of OxCal (see S2 File), and (ii) a χ^2^ fit approach that weights the differences according to the respective measurement errors (e.g. [28, 32, 33]):

$$\chi {\left(x\right)}^{2}=\sum_{i=1}^{n}{\left(R_{obs}-R_{exp}\right)}^{2}/({{\sigma R}_{obs}}^{2}+{{\sigma R}_{exp}}^{2})$$

Where R_obs_ is the NOC ^14^C value (or observed data) and R_exp_ is the expected ^14^C value (IntCal20), and σR_obs_ and σR_exp_ are the respective measurement errors on each of R_obs_ and R_exp_. The χ^2^ value should become minimal for the correct fit year: *x*. (Note the sign is incorrect in the top line of the equation in [33]).

**Table 3:** The relative year (RY) dates of the Noceto (NOC) tree-ring samples cut for ^14^C dating from NOC-12A and 14A with Groningen (GrM) lab codes, δ^13^C values and measured ^14^C ages.

Sample	RY begins	RY ends	GrM-	Treatment	δ^13^C ‰	SD	^14^C Age BP	SD
NOC-14A	995	999	17548	ABA-B	-27.10	0.15	3380	25
NOC-14A	1000	1004	17645	αc	-27.65	0.15	3375	25
NOC-12A	1050	1054	11242	αc	-25.47	0.05	3320	25
NOC-14A	1050	1054	13697	ABA-B	-26.80	0.05	3274	15
NOC-14A	1060	1064	13749	ABA-B	-27.08	0.05	3332	15
NOC-12A	1080	1084	11243	αc	-25.83	0.05	3360	25
NOC-14A	1080	1084	13750	ABA-B	-25.11	0.12	3332	15
NOC-14A	1100	1104	13751	ABA-B	-23.97	0.12	3317	15
NOC-12A	1120	1124	11331	αc	-25.83	0.05	3227	14
NOC-12A	1120	1124	13696	ABA-B	-25.94	0.05	3282	15
NOC-14A	1120	1124	13752	ABA-B	-25.97	0.12	3260	15
NOC-14A	1120	1124	17679	αc	-26.50	0.15	3255	25
NOC-14A	1140	1144	13754	ABA-B	-25.94	0.12	3263	15
NOC-12A	1160	1164	11275	αc	-25.13	0.05	3330	25
NOC-12A	1160	1164	17406	αc	-25.13	0.05	3250	25
NOC-14A	1180	1184	13755	ABA-B	-25.83	0.12	3222	15
NOC-14A	1200	1204	17737	αc	-26.92	0.15	3160	35

ABA-B = ABA-bleach pretreatment, αc = alpha-cellulose pretreatment (for Groningen methods, see [24]). We note that GrM-11275 is an unexplained outlier (too old) and is not plotted or used. The NOC-12 RY1160-1164 sample (not NOC-14, correcting typo in [25] at S1 Table) was subsequently re-prepared and re-dated as GrM-17406. We employ this date instead.

In previous work it was observed that the Noceto ^14^C dates indicated an apparent ^14^C difference versus the IntCal13 calibration curve [25]. The subsequent revision of the northern hemisphere calibration record in the period 1700–1500 BCE, as published in the IntCal20 calibration curve–with the addition of hundreds of new modern AMS ^14^C measurements [29] has largely removed this issue with respect to the Noceto series [34]. A test using the OxCal Delta_R function [27] to test for and to assess whether there is any systematic difference between the Noceto time series (all data, n = 16: Table 3 and model structure in S2 File) and the IntCal20 calibration curve using a neutral prior (0 ± 10 ^14^C years), indicates no substantive offset: μ ~2.2 with σ ~5.9/6.0 ^14^C years across several model runs (and even less, μ ~1.2 and σ ~5.8/5.9 ^14^C years, if the analysis excludes the two outliers: see discussion below, so n = 14). This circumstance suggests that the previously observed difference in the Noceto case was largely due to problems with the previous calibration curve measurements and in line with a general observation in several instances that modern AMS ^14^C measurements yield ^14^C ages that are slightly older than legacy conventional results [25, 34–37]. The comparison, now, of the modern AMS ^14^C age estimates on the Noceto tree-ring samples versus the IntCal20 calibration record largely based on similar technology for the relevant time period removes this issue. Otherwise, as observed, no substantive growing season offset is to be expected for these northern Italian oak trees, versus the trees providing the ^14^C data for the IntCal20 record, which all have relatively similar and overlapping growing seasons, although climate responses generally vary north versus south of the Alps, broadly later April/start May through late August/mid-September [25, 38, 39]. Hence we place the Noceto time series versus IntCal20 and regard this correlation as likely yielding an approximately accurate calendar position.

Seven ^14^C dates are also available from previous work on materials from the upper and lower tanks at the Noceto site (Table 4). These dates can help elucidate the use periods of the tanks, especially when combined with both the stratigraphic information and the dates available for the use of the timbers. We employed these seven ^14^C dates via a Bayesian chronological model that combined them with the stratigraphic order information, older to more recent (lower tank construction > lower tank use > upper tank construction > upper tank use), and including the calendar date estimates for the likely felling /construction dates for the Upper Structure (RY1206 and RY1218) derived from the tree-ring sequenced ^14^C wiggle-matching of the samples from NOC-12 and 14. We assume the samples from the use of each tank are a random sampling from within the overall use period of that tank (versus, in the case of the upper tank where we have several samples, all deriving from a single event, or all deriving from the beginning or end of the use of the tank). We again employed the OxCal software [26–28] and the IntCal20 ^14^C calibration dataset [29]. The OxCal General Outlier model [27] was applied to test for and to down-weight outliers. The OxCal runfile is listed in the S3 File.

**Table 4:** ^14^C dates run on other samples from the Noceto upper and lower tanks.

Lab ID	Noceto ID	Material	^14^C Age BP	SD	Calibrated Dates BCE
					95.4% probability
LowerTank					
UGAMS-29350	US555	Dogwoodberry stone	3080	25	1415–1270
UGAMS-29349	US172a	Oak gall	3160	25	1499–1396 (94.5%), 1331–1327 (0.9%)
WoodUpperTank					
Poz-25259	US148	Wood	3225	35	1600–1586 (1.6%), 1543–1418 (93.8%)
UpperTank					
Poz-23426	US5002	Bone collagen	3125	35	1495–1477 (4.4%), 1458–1289 (91.0%)
Poz-19307	US5003	Hazel shell	3085	35	1430–1260 (95.0%), 1240–1236 (0.5%)
Poz-19036	US66	Hazel shell	3075	35	1423–1258 (93.0%), 1245–1230 (2.4%)
Poz-25258	US81	Dogwoodberry stone	3115	35	1492–1481 (1.8%), 1451–1279 (93.7%)

Data from [4] and in part previously unpublished. Calibrated age ranges (no modelling) for each ^14^C date from IntCal20 [29] using OxCal [26] version 4.4.2 are listed.

## Results

### Tree-ring analysis

**Fig 11:**
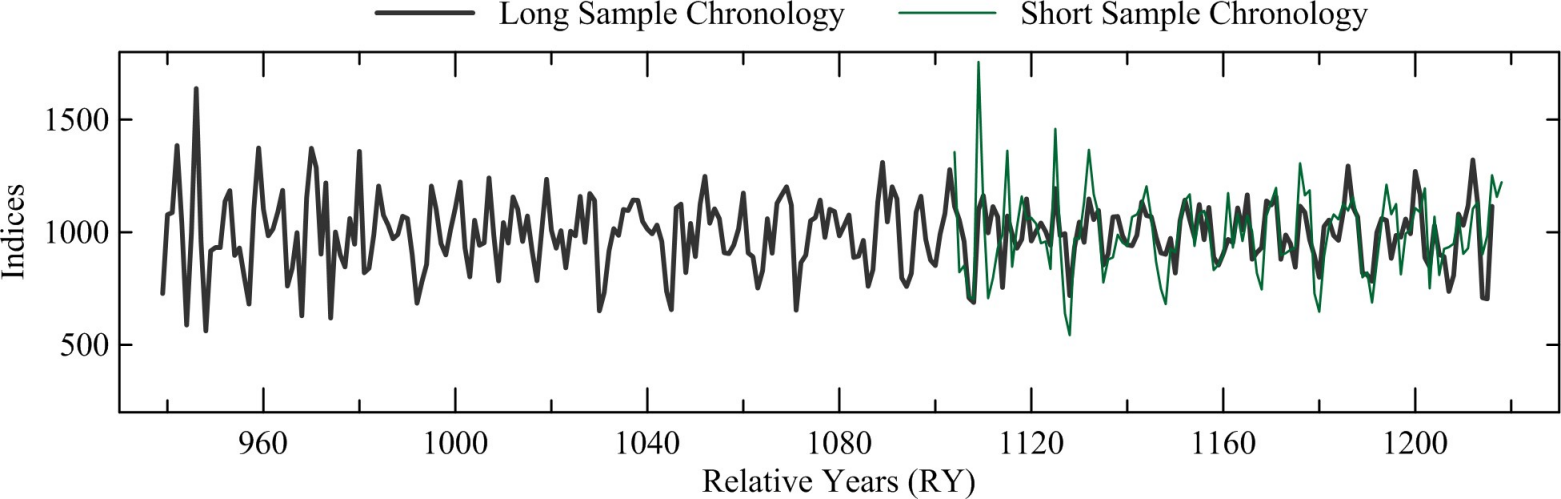
Comparison of the tree-ring widths of the Noceto oak chronology composed of the 9 long sequences with the Noceto oak chronology comprising the 5 short sequences. Supporting statistics are t = 7.42, r = 0.58, tr = 0.67, all with *p* < 0.01, with an overlap of 113 years.

**Fig 12:**
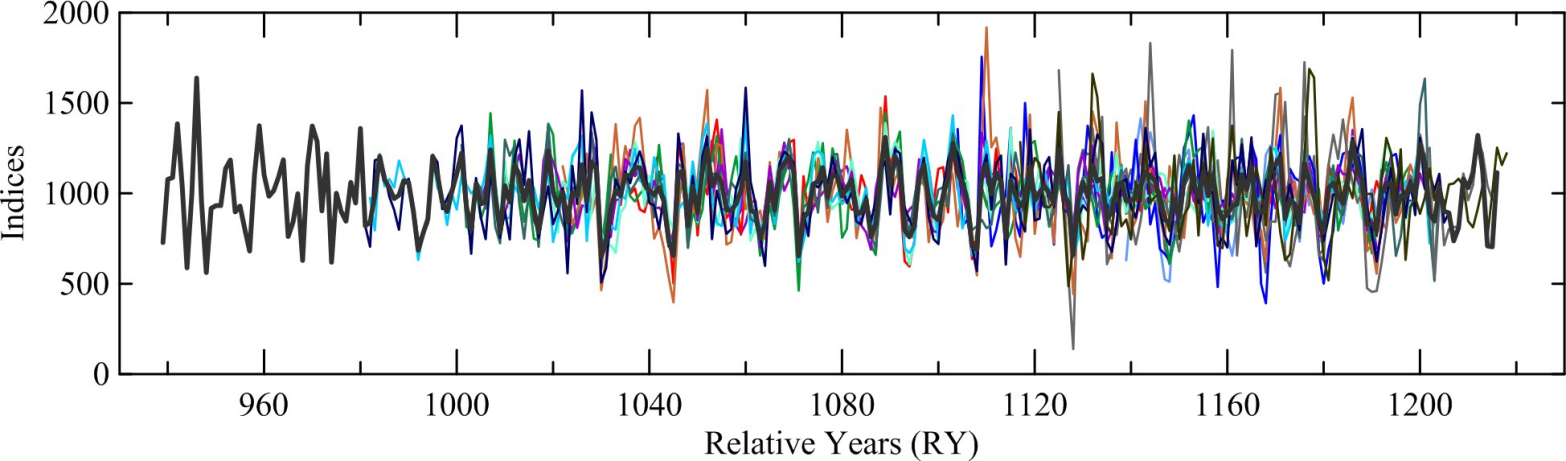
The combined Noceto tree-ring width chronology comprising 14 constituent elements.

The ring counts (measured rings) of the 25 oak samples range from 71 to 281, with a ring count of over 150 in 19 samples (Table 2). The comparison of ring-width sequences of the oak samples indicated that 5 trees are represented by multiple samples, with 16 samples from these 5 separate trees (Table 2). The sequences from samples in the same tree were combined into one data set for each tree. The remaining 9 samples represent 9 separate trees, with a total of 14 oak trees likely represented in this sample collection. Of the 14 tree sequences, 9 trees, all with over 150 ring count, have very similar ring-width patterns, both visually and statistically, and were relatively dated to each other and combined into a long relatively-dated chronology, 278 years in length with relative years (RY) 939–1216. The other five trees, with ring counts below 150, were relatively dated by successfully matching the growth patterns in each tree to the long chronology and combining them into a separate chronology, 115 years in length, at RY1104–1218. The visual match and supporting statistics of t = 7.42, r = 0.58, and tr of 66.5% (Fig 11) between the long and short chronology confirmed that the relative placements of the short sequences are secure. All samples were then combined into the overall Noceto Chronology, 280 years in length, RY939–1218 (Fig 12).

The date and completeness of the outermost rings of those oak samples with sapwood, and especially the samples with possible waney edges, were then inspected to estimate the relative date of felling for each tree. Bark was absent in all oak samples and only one sample, NOC-27, had a waney edge with its outer ring complete and continuous around at least a quarter of its circumference at the A (first measured) radius and in the outer rings of 4 other radii investigated around the sample. RY1218 in NOC-27 is thus likely the felling date. For all other samples we must be less certain. The outer ring may be at the waney edge in a number of cases, but its lack of continuation around the circumference, plus the natural degradation of outer rings, whether from the susceptibility of sapwood to drying, insect damage, or from the long period of burial, all leave the possibility that one to a few rings are missing. This especially applies where the number of sapwood rings is lower than might be anticipated. It is conspicuous that six tree samples have last extant tree-rings RY1204–1206 and another three trees have last extant tree-rings RY1216–1218 (including one with waney edge). This might suggest both groups are close to exhibiting likely common felling dates, e.g., a first group around or just after RY1206 and a second group around RY1218. A reasonable hypothesis could be that this circumstance represents felling dates relevant, respectively, for the lower and then upper tanks at Noceto. In this regard, it is important to note the observation, made during the conservation and restoration of the Noceto timbers, that several of the wooden elements originally from the older lower tank and structure were re-used in the building of the more recent upper tank.

**Fig 13:**
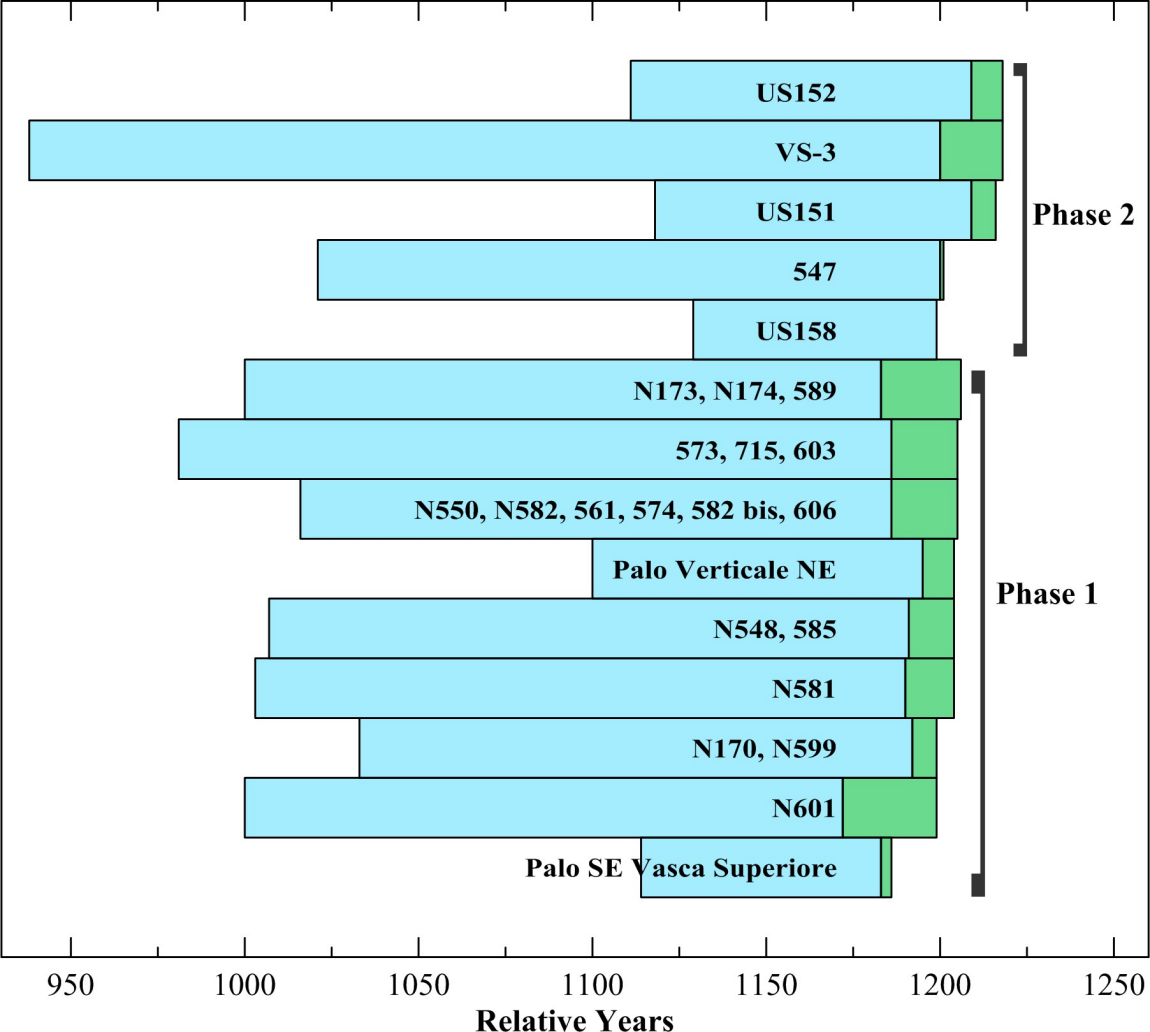
Bargraph showing the crossdated placements of the timbers from the two phases identified among the Noceto samples.

We can seek to refine felling estimates by considering the expected number of oak sapwood rings. A review of European oak data reported an average of 13.23 sapwood rings (median 15) for Italy and a range in total of 5–38 sapwood rings and a 95% range of 5.66–30.93 sapwood rings [38]. A recent study on oaks in the adjacent Pannonian Basin reports similar data with an average of 13.3 sapwood rings and an overall range of 5–32 sapwood rings [40]. The latter study observes the expected relationship where the number of sapwood rings in oak tends to increase with tree age [41, 42]. As in Table 2, we consider what appear–subjectively–to be two groupings within the dataset (Fig 13).

#### Phase 1

For the group with last rings RY1204–1206, five of the six trees have >150 total rings (mature trees) and the number of sapwood rings range from 13–23. In principle, given the above expected ranges, there is no reason to expect many missing sapwood rings. The two trees both with 19 sapwood rings (NOC-6,8,12,15,16,20 & NOC-14,19,21) have a last extant ring at RY1205 and the tree with 23 sapwood rings (NOC-3,4,18) has a last ring at RY1206. The latter especially might be a felling date, leaving 1 sapwood ring missing from the RY1205 trees if the group were all felled in the same year. The trees ending RY1204 have 13 (NOC-5,17) and 14 (NOC-7) sapwood rings and might most easily be missing perhaps 2 outermost sapwood rings if all trees were felled RY1206. NOC-25 with only 105 rings in total also ends RY1204 with 9 sapwood rings. As a less mature tree a lower number of sapwood rings would be expected and to be missing only ca. two sapwood rings is plausible. Thus, the group identified in Table 1 as Phase 1 might well all reflect felling in (or very close to) RY1206. Tree NOC-3,4,18, with 207 total rings ending RY1206, has 23 sapwood rings, and is a mature but not very old oak. Hence the latest plausible felling date is likely within the 95% range for Italian oak sapwood values (so no more than ca. 31 sapwood rings), and thus at a maximum its felling date is likely no more than ca. 8 years later at the very most (RY1214). Even if this scenario applied, the date is still before the group of trees (Phase 2 in Table 1) felled likely RY1218. The most economical scenario is that NOC-3,4,18 belongs with the ca. RY1206 group despite no visible waney edge, likely due to degradation/preservation circumstances. Therefore, it seems likely there are two phases of felling, and thence likely construction work, involved in the Noceto structure on the basis of the samples investigated in this work.

All of the Phase 1 samples except NOC-3 and 4 are from the lower tank structure and thus might be regarded as belonging to this older tank’s construction. NOC-3 and 4 appear to represent the same tree as NOC-18 from the lower tank. Thus it is very plausible that NOC-3 and 4 were originally lower tank elements re-used in the subsequent upper tank. The Phase 1 cutting date around RY1206 appears likely to offer a date for the lower tank.

#### Phase 2

The second group of trees have a waney edge case for RY1218 (NOC-27). This tree was not very old, total 108 rings with 10 sapwood rings. The other tree with a last extant ring at RY1218, NOC-22, in contrast has 281 rings overall and 18 sapwood rings. This value suggests that its last extant ring is in reality the waney edge (just not preserved sufficiently to be clearly recognizable), but alternatively it could have been felled a few years later. NOC-26 with a last extant ring at RY1216 has only 99 years (rings) in total and so its 7 sapwood rings may plausibly only be missing ca. 2 rings and it could again be a RY1218 felling. Each of these samples (NOC-22, 26, 27) derive from the upper tank. NOC-11 is a mature tree (181 rings) with just 1 sapwood ring present ending RY1201. It would be plausible that this tree is missing ca. 17 sapwood rings and so could also represent a RY1218 felling and the upper tank–but there is a complication: NOC-11 comes from a lower tank context. Barring unexpected ideas of some renewal or intervention into the lower tank remains as part of constructing the upper tank, this sample might just have had very few sapwood rings (e.g., ca. 5 and represent a RY1206 felling). We have no way at present to resolve this complication. NOC-28 is a younger tree, 71 rings present ending RY1199. It was found as part of the upper tank. NOC-28 has no sapwood rings and its outermost extant ring may be at the heartwood-sapwood boundary where the change in wood density makes it more susceptible to breakage than in other parts of the xylem. With a minimum of 5 sapwood rings it could in fact be part of Phase 1 (and if so re-used in upper tank), but, it is also uncertain whether the outer preserved ring is necessarily the heartwood/sapwood boundary. If this instead reflects carpentry activity, heartwood rings may also be missing. In that case, it is more likely part of Phase 2. We note the uncertainty and have opted to place NOC-28 tentatively in Phase 2.

Overall, as shown in Fig 13, there seem to be two phases of felling and thus likely carpentry and construction represented about a dozen years apart, one around or just after RY1206 that likely dates the felling for and construction of the lower tank, and one around RY1218 that likely reflects the felling for and construction of the upper tank. Allowing that it is likely use of timber in a construction project such as this was within 0–1 year of felling with the timber still green and easy to work, this implies a ca. 12-year period between the construction of the lower tank and the construction of the later upper tank.

The same methods of crossdating and identifying outer rings were applied to the three elm samples, but resulted only in identifying that two are from the same tree and contain a waney edge (NOC-13 & NOC-24; Table 1). However, its 71-year ring count and the 25 rings in NOC-1, plus the lack of additional elm samples, did not allow any further dendrochronological analysis of the elm material.

The dendrochronological results from the study of the oak samples place nine represented trees into the Phase 1 chronology, RY982–1205, with an incomplete and unmeasured ring at RY1206, and five oak trees into the Phase 2 chronology, RY939–1218, ending with a complete and measured ring and waney edge. The Phase 1 and 2 chronologies were combined into the Noceto Oak Chronology of 280 years, RY939–1218 (Figs 12 and 13). Unfortunately, no long absolutely-dated oak reference chronologies from this period or region exist to our knowledge at this time, and therefore ^14^C dating was necessary to absolutely date the chronology in order to obtain the felling-construction dates for the two phases.

### Radiocarbon wiggle-match dating

**Fig 14:**
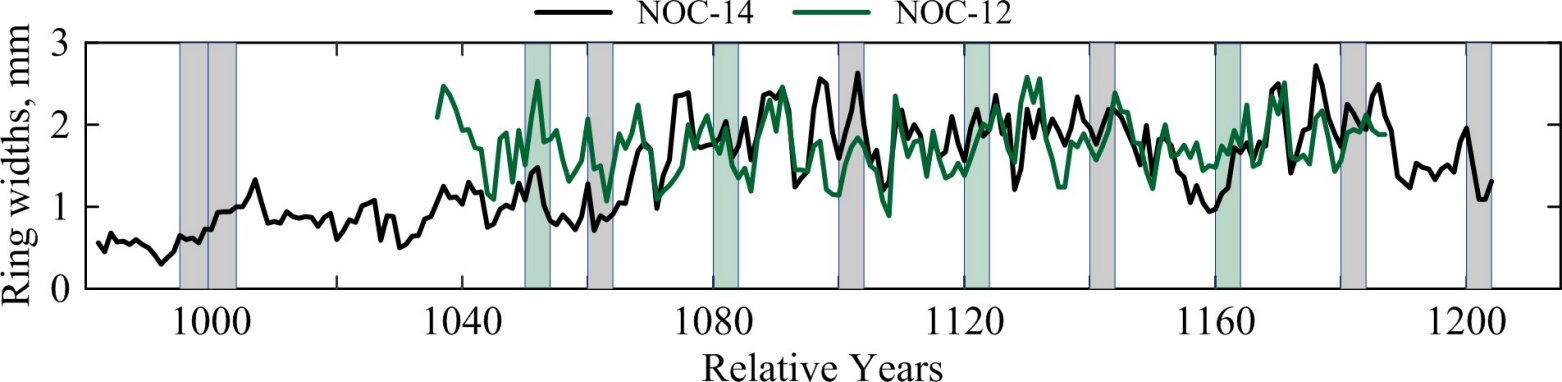
The tree-ring width patterns for the NOC-12 and 14 samples showing where the segments of NOC-12 (green bars) and NOC-14 (gray bars) were taken for ^14^C dating.

To obtain a well-defined near-absolute date for the lower and upper Noceto tanks we employed ^14^C ‘wiggle-matching’ [28, 31–33]. This takes the calendar spacings (tree-ring spacings) of the ^14^C dates to create a fixed time series that is then best fitted against a reference known-age ^14^C calibration dataset (in this case IntCal20 [29]). Eleven 5-year segments were dissected from two samples from two different trees (NOC-12 and NOC-14) (these ^14^C dates and the NOC-12 and NOC-14 crossdate have been discussed previously [25]) (Fig 14). We chose samples that crossdate, have high ring counts with the volume of material needed for ^14^C analysis, and have definitely not been conserved/treated with PEG. The ^14^C dates are listed in Table 3.

**Fig 15:**
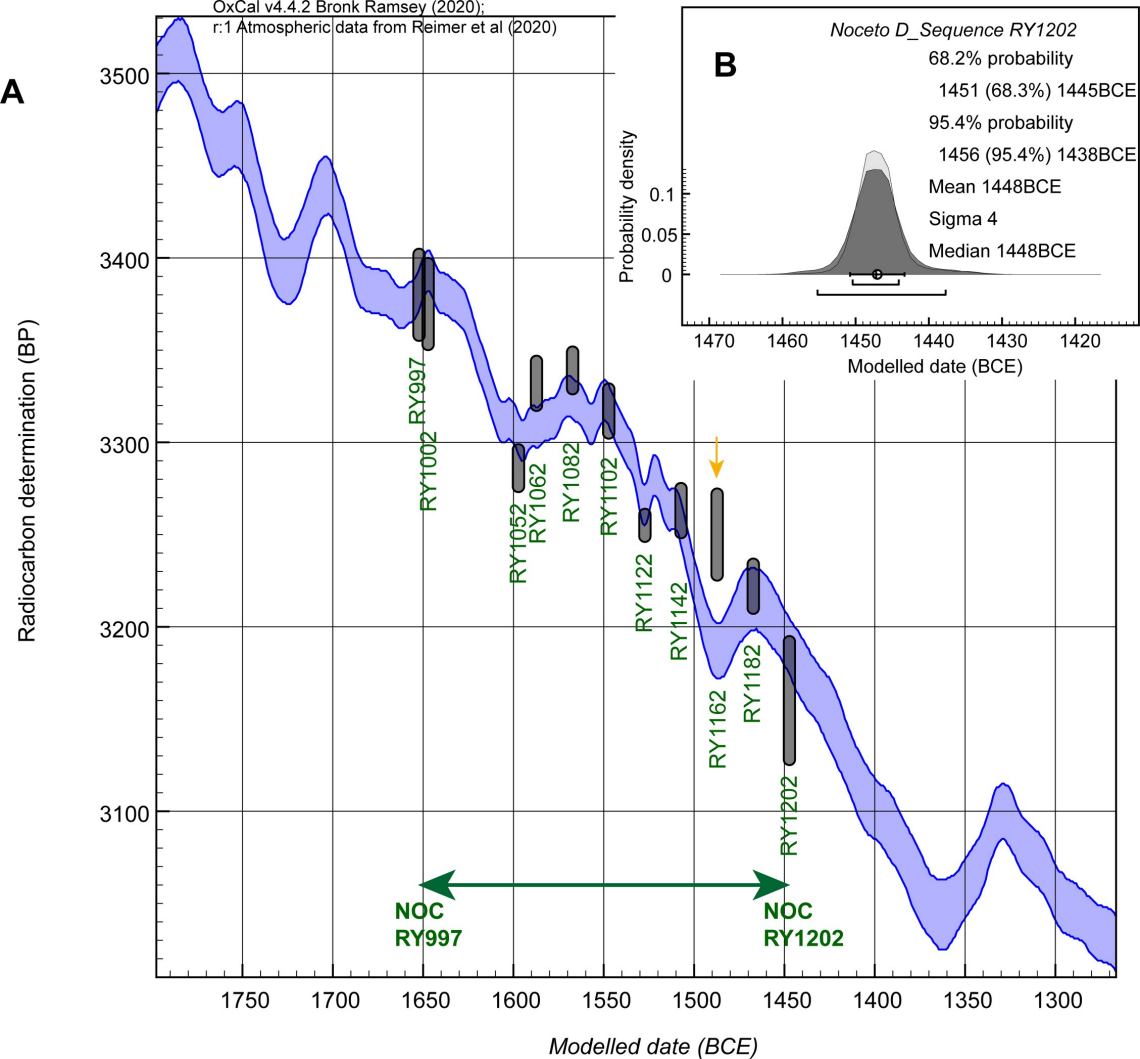
OxCal wiggle-match of the 11 dated NOC elements against the IntCal20 ^14^C calibration curve. A. ^14^C dates (or weighted average ^14^C values) for NOC dated elements (each 5 tree-rings/years) shown at 68.3% probability (Y axis) against the IntCal20 calibration curve (68.3% probability band) [29]. The date centered RY1162, indicated with arrow, is a minor possible outlier (outlier probability ~12%). B. The fit probabilities for the latest ^14^C dated element: RY1202. Data from OxCal [26–28] version 4.4.2.

**Fig 16:**
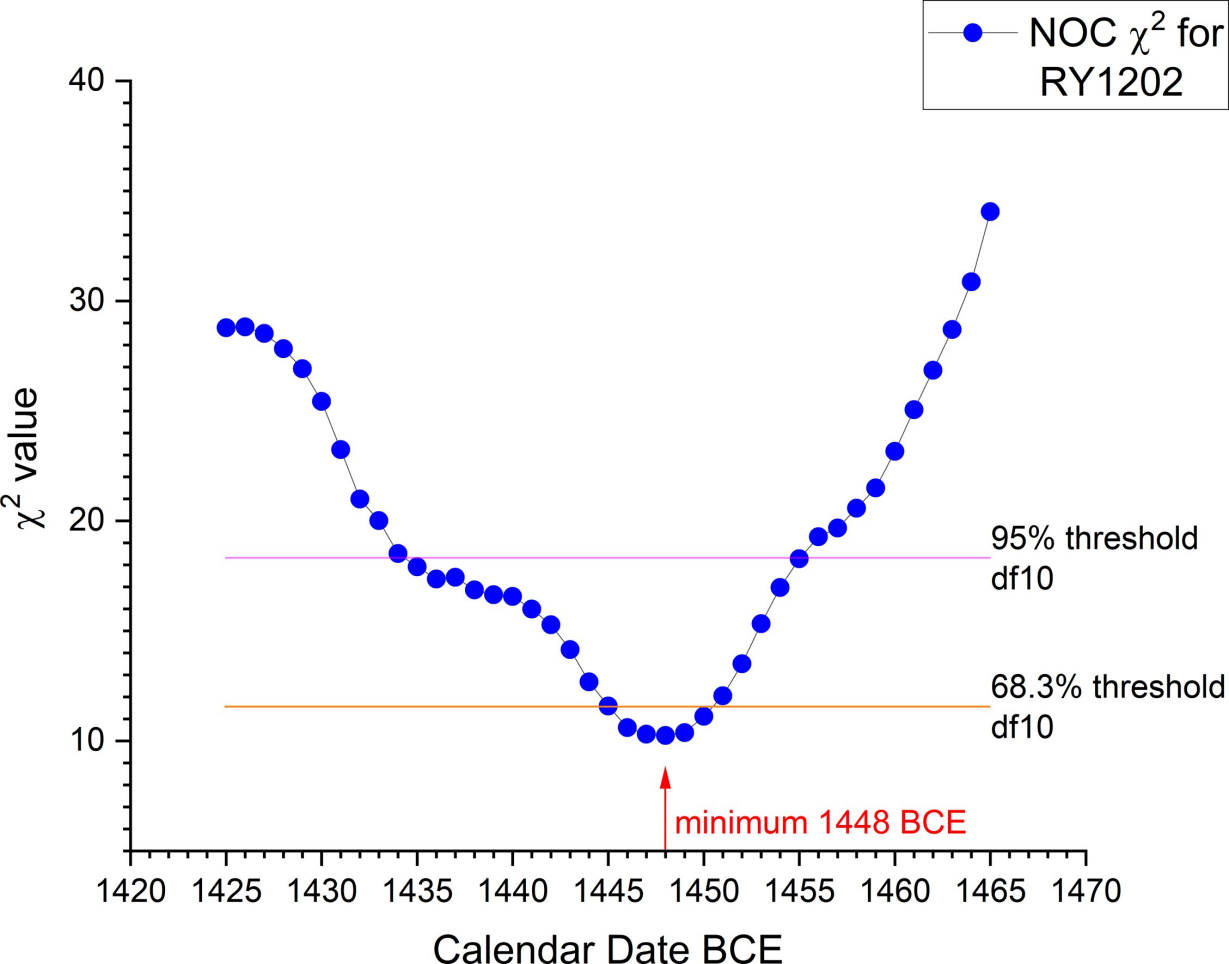
χ^2^ fit function for the NOC wiggle-match against the IntCal20 ^14^C calibration curve [29] expressed in terms of the placement of RY1202 (the last ^14^C dated element).

The results of the wiggle-matching are shown in Figs 15 and 16. The OxCal model achieves A_model_ = ~71 and A_overall_ = ~74, both greater than the satisfactory threshold value of 60. In terms of the last dated mid-point, NOC RY1202, the median or mean of the OxCal fit range is 1448 BCE and the error on the mean is ±4 years. The OxCal 68.3% highest posterior density (hpd) range is 1451–1445 BCE, and the 95.4% range 1456–1438 BCE. The RY1162 value (GrM-17406) offers poor individual agreement with the model (A = ~21 < 60), and has a ~12% outlier probability. One date within the R_Combine for RY1122, GrM-11331, is also flagged as a larger outlier (~49% probability). Removing these two dates, however, makes no real difference to the wiggle-match. The A_model_ value goes up to ~138 and A_overall_ to ~137, but the median or mean placement for the wiggle-match remains the same: 1448 BCE for RY1202 (error now ±3 years). The 68.3% hpd range is now 1451–1444 BCE, and the 95.4% range 1454–1441 BCE. We note that changing the curve resolution to 5 years also achieves very similar placements for both cases. The all-data model typically finds a best fit 0–1 years more recent than shown in Fig 15 (RY1202 placed mean 1447 BCE, median 1448 BCE) and the revised model excluding the two outliers typically achieves a best fit identical to that shown in Fig 15.

The minimum value from the χ^2^ fit is also 1448 BCE (χ^2^_red_ = 1.03). The 95% χ^2^ fit range (those values < χ^2^ df10 = 18.31) is 1455–1435 BCE, and the 68.26% χ^2^ fit range (those values < χ^2^ df10 = 11.53) is 1450–1446 BCE. These χ^2^ ranges are both very similar to the OxCal ranges.

We thus place the Noceto chronology with RY1202 at ~1448 BCE. Based on the revised model (excluding the two outliers), the error around the mean of the distribution is 3 years and the difference from the 68.3% hpd range is +3/-4 years. We therefore regard a realistic and conservative 68.3% error estimate as ± ≤4 years. This error estimate (or those around 1448 BCE for the 95%/95.4% ranges cited above) applies to all dates cited for the Noceto chronology. The start of the Phase 2 chronology and overall Noceto tree-ring chronology, RY939, is therefore dated ~1711 BCE, and the start of the Phase 1 chronology, RY982, is ~1668 BCE. The last extant tree-ring in the Phase 1 chronology, RY1206, is placed ~1444 BCE. The last extant tree-ring in the Phase 2 chronology, and for the overall Noceto chronology, RY1218, is placed ~1432 BCE. In particular, NOC-27 RY1218 has waney edge present, and so likely represents a felling date. Thus ~1432±4 BCE likely dates the felling for, and construction of, the Noceto upper tank. Other Phase 2 samples allowing for plausible missing sapwood were likely felled about the same time. NOC-22 could be at its final ring (RY1218), but a waney edge was not observed. This may reflect preservation. It might also indicate felling another year (or two) later. As noted above, the Phase 1 group seems likely to represent a felling episode about a dozen years earlier and to represent the felling for, and construction of, the earlier lower tank at Noceto placed ~1444±4 BCE.

### Other radiocarbon dates

**Fig 17:**
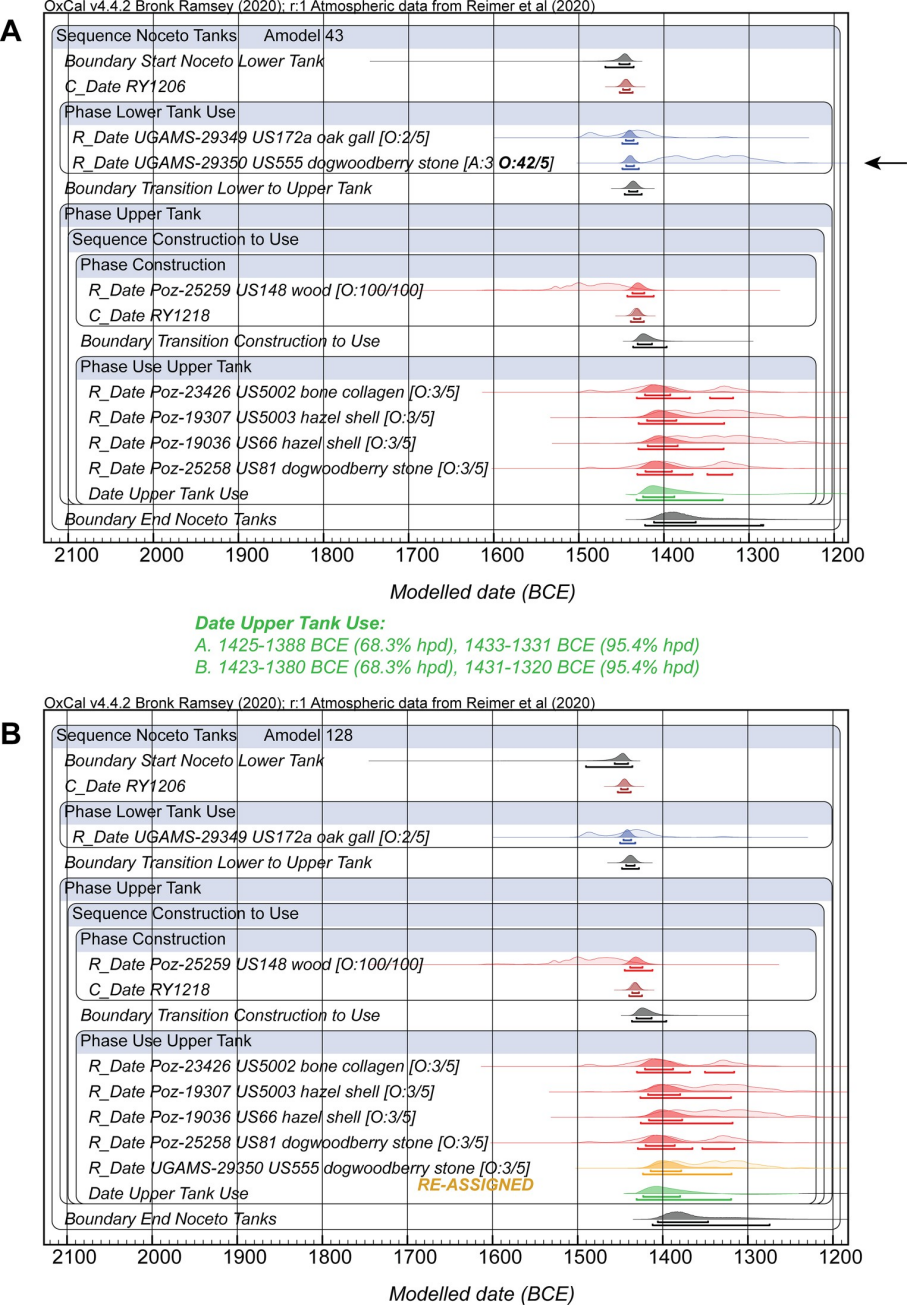
Bayesian chronological model combining (i) the wiggle-match date for lower tank felling-construction, (ii) ^14^C dates on short-lived sample(s) associated with the use of the Noceto lower tank, (ii) a ^14^C on wood of the upper tank and the wiggle-match date for the upper tank felling-construction, and then (iii) ^14^C dates on short-lived samples associated with the use of the upper tank. The OxCal General Outlier model is applied to the ^14^C dates on short(er)-lived samples, the OxCal Charcoal Outlier model is applied to the date on a wood sample (unknown what tree rings dated and we assume some in-built age likely) [27]. A. All data with UGAMS-29350 a clear outlier (too recent, indicated by arrow)–perhaps a sample that should belong with the upper tank use period? B. The model re-run after re-assigning UGAMS-29350 to the upper tank period of use. The results are very similar. Data from OxCal [26, 27] version 4.4.2 using IntCal20 [29] with curve resolution set at 1 year. The lines under each distribution indicate the modelled 68.3% and 95.4% hpd ranges respectively.

Seven ^14^C dates are available from previous work on materials from the site and are in part previously unpublished: see Table 4. A Bayesian chronological model combining these dates with the stratigraphic order information and including the calendar date estimates for the likely felling-construction dates for the lower tank (RY1206) and upper tank (RY1218) at 1444±4 BCE and 1432±4 BCE respectively is shown in Fig 17A. One of the lower tank use dates, UGAMS-29350, is conspicuously too recent to belong to the lower tank and instead appears likely to relate to the use of the later upper tank (and so appears to be a mis-association in terms of find context reported). It has an ~42% outlier probability with the OxCal General Outlier model [27] (and an OxCal individual Agreement value of ~3 < 60) and is accordingly down-weighted in the model (and largely explains the poor A_model_ and A_overall_ values, <60, for the initial model). The ^14^C date on ‘wood’ from the upper tank, Poz-25259, not surprisingly indicates a date range mainly including years older than the felling estimate–this is entirely plausible since the wood dated was likely older (‘old wood’ effect unless outermost tree-rings) than the waney edge on NOC-27 and perhaps by some decades or more depending on exactly which tree-rings were sampled for this date (unknown). The OxCal Charcoal Outlier model [27] approximately allows for this (Fig 17A and 17B).

**Fig 18:**
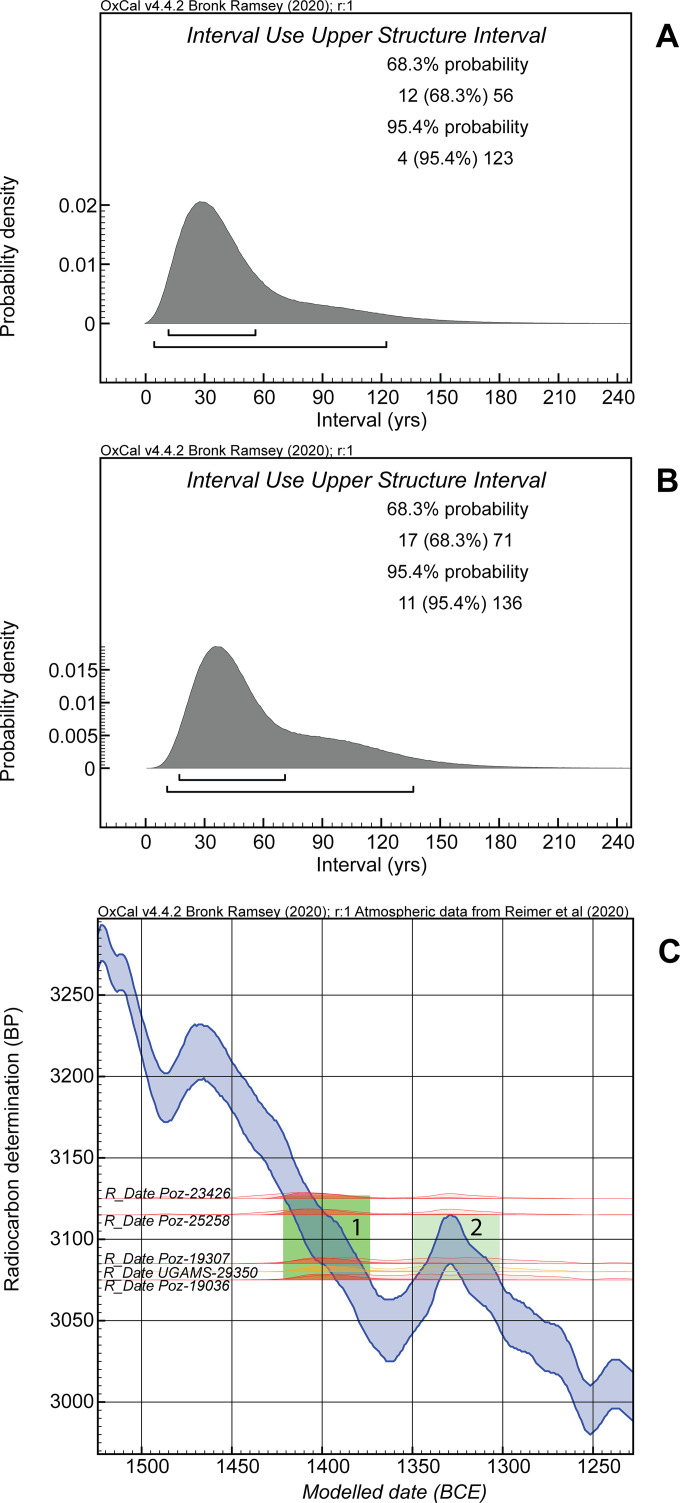
The results of an OxCal Interval query applied to the Noceto upper tank use phases in Fig 17A and 17B to estimate the period of time after construction through end of use of the upper tank and placement of the upper tank use period versus the ^14^C calibration curve. A. The use period from the Fig 17A model with all data. B. The use period from the Fig 17B model with UGAMS-29350 re-assigned. C. The five ^14^C dates from the upper tank use period (Fig 17B model) shown as placed against the calibration curve. The hollow distributions indicate the unmodelled calibrated calendar age probabilities; the solid distributions indicate the modelled calendar age probabilities. The area labelled as 1 indicates the most likely range for these data and a shorter period of use for the upper tank; the area labelled as 2 is the alternative placement due the wiggle in the calibration curve.

The other ^14^C dates on short/shorter-lived material from the use of the upper tank can provide some indication of the period of time over which the upper tank was in use following its construction ~1432±4 BCE. The similar ^14^C ages for the four samples suggest a relatively short overall period is represented. Indeed, the four ^14^C measurements, or the five if UGAMS-29350 is re-assigned, could be consistent with the hypothesis of representing estimates of the same ^14^C age [30] (respectively 3100±18 BP or 3093±15 BP). However, we assume they represent more than one event, and are a random sample from the use-period of the upper tank. The model in Fig 17A places the boundary demarcating the overall end of the upper tank phase at 1412–1363 BCE at 68.3% hpd (1423–1284 BCE at 95.4% hpd). Thus it appears there was a relatively short overall lifetime for the usage of the upper tank. An OxCal Interval query for the upper tank phase duration yields ~12–56 years at 68.3% hpd (or 4–123 years at 95.4% hpd) (Fig 18A).

If the model is revised, moving the likely out of context UGAMS-29350 into the upper tank use phase where it likely really belongs and perhaps as one of the latest elements, then the A_model_ and A_overall_ values become good each around ~128, and the likely age ranges determined stay very similar (see Fig 17B). The overall upper tank use period is calculated in this revised model as very slightly longer at 17–71 years at 68.3% hpd and 11–136 years at 95.4% hpd (Fig 18B). This revised use period ends (End Noceto Tanks Boundary) most likely by 1406–1347 BCE (68.3% hpd, 1412–1275 BCE at 95.4% hpd).

## Discussion

### The two tanks at Noceto

Most of the wood samples recovered from the lower tank belong to Phase 1 (likely cutting date around RY1206 = 1444±4 BCE) as identified above, the oldest tree cutting or felling period. NOC-11 (N 547) is the only exception as noted above. Possible explanations are either that this was a tree with very few (e.g. 5) sapwood rings, or, alternatively, that it was a subsequent insertion into the lower tank timbers made for some reason at later time and perhaps as part of the work undertaken in building the upper tank a dozen years later. The upper tank seems defined, in time, by timbers with a felling date around RY1218 = 1432±4 BCE. A number of the upper tank boards/planks were, however, instances of re-use of lower tank (Phase 1) wood. This is not surprising given the availability of this wood at the site and the effort involved in wood procurement and working. During the reassembly of the archaeological materials from the monument in the Noceto Museum, it was observed that a number of the boards of the upper tank were apparently recovered and recycled from the lower tank after its collapse. Such re-cycling appears to have focused on the boards/planks used for the horizontal (containing) elements. The beams and poles employed to form the key structurally integral basal framework of the upper tank all belong to Phase 2 –when the coordinated new, and revised, upper tank was constructed. These beams have different dimensions (longer) and are thus incompatible with the beams of the lower structure, and therefore cannot have been recovered from it, and must have been cut and prepared specifically for the construction of the upper tank. The two building phases detected in the dendrochronological investigation of woods likely refer to the two different moments of the construction of the monument: Phase 1 represents the wood procurement to build the lower tank, whereas Phase 2 represents procurement of a further set of trees to build the upper tank.

### Dating and the archaeological chronology of the site

The dates of the two felling and construction periods suggest an approximate near-absolute chronological framework. The building of the lower tanks of the Vasca Votiva likely happened at ~1444±4 BCE (RY1206), whereas the construction of the upper tank started about 12 years later, likely ~1432±4 BCE (RY1218). There is a good correspondence between the dendrochronological dating results and the chronological-typological analyses of the pottery assemblage found at the bottom of the lower structure pointing to the advanced Middle Bronze Age 2 (BM2B) [3, 9, 10]. This age suggests a chronological association with the advanced BM2 to BM3 phase of the Montale site [1, 43]–a nearby Terramara settlement, where the BM2 to BM3 transition is placed ~1450 BCE. Conversely, the construction of the upper structure, which is dated by the waney edge present on one of the Phase 2 timbers (NOC-22) at about 1432±4 BCE, corresponds to a later pottery assemblage attributed to the BM2B –BM3 transition that occurred in the late Middle Bronze Age. The upper structure remains in use for likely a relatively short period. If we assume that we should re-assign the UGAMS-29350 sample to the upper tank use period, and if we assume that the five dated samples from the upper tank use represent a random sample from the whole of the use period of the tank, then according to the Bayesian model (a date query for the upper tank use phase) (Fig 17B) this covers the interval between 1423–1380 BCE (68.3% hpd, 1431–1320 BCE at 95.4% hpd). This chronological attribution conforms well with the chronological-typological analysis of the pottery from the upper part of its infilling, which belongs to the full BM3 [10].

Determining the length of use assigned to the upper tank is complicated by the shape of the ^14^C calibration curve. The 4 or 5 ^14^C dates from the use of the upper tank belong to a period with a substantial ‘wiggle’ in the calibration curve (Fig 18C). They could all and most likely fit in the period from ~1420–1380 BCE, or they could, with less probability, fit later in the period either side of the wiggle centered ~1329 BCE. The most economical and probable interpretation is the shorter scenario (as indicated by the most likely 68.3% hpd ranges in Fig 18A and 18B). This is also consistent with the pottery from the upper part of the infilling of the upper tank which does not indicate a date later in the 14^th^ century BC [10].

The cause of the collapse of the lower structure remains uncertain. We can suggest that some mistakes in the design of the structure or its building technology may have hampered its stability and reduced its ability to resist the lateral stress from the walls of the building pit. But this is only a guess. An alternative hypothesis that cannot be excluded is that some sudden and unexpected event, for example an earthquake, may have triggered the collapse of a significant part of the lower structure [9]. The possibility of an association with a seismic event is potentially likely, since the whole area around Noceto is tectonically active. In either case, the use of a different building technology in the construction of the upper tank suggests a deliberate effort to counter previous problems—whether a simple collapse through poor design/engineering leading to structural weakness, or collapse associated with a seismic or other event.

The date of the Vasca Votiva at Noceto is notable. It is contemporary with the transition from the BM2 (BM2B) to BM3 periods of the Middle Bronze Age of northern Italy or to the very beginning of the BM3 phase. BM3 is a time marked by societal shifts and major changes in settlement pattern and in settlement structure and organization within the wider region of influence of the Terramare culture, the beginnings of new funerary practice (cremation starts to occur and subsequently becomes the dominant funerary practice), and likely associated changes are observed from this time in both environment/climate and the nature of land use (increased deforestation to support the expansion of cultivation and pasture) [5, 11, 44]. The date raises the question of whether and how the Vasca Votiva at Noceto and the associated ritual practices were involved in the development and expression of what were becoming a set of new life-ways (landscape, task-scape, social-scape, political-scape) in the region. The Terramare culture, with villages set within banks and moats in the alluvial plain of the Po river, centered around irrigation agriculture, along with pastoralism, in at least one case (close by Montale) likely intensive textile production, and long-distance trade [43, 45].

The Vasca Votiva at Noceto appears to materialize and represent part of a set of evidence for ritual practice and belief associated with water and its procurement, storage, and use [2]. Such water availability and its manipulation appear to form part of the basis to the growth and success of the Terramare culture—whereas later towards the end of the second millennium BCE (13^th^-12^th^ centuries BCE) increasing aridity appears linked with its decline and collapse [5]. We suggest a primarily non-utilitarian role for the Vasca Votiva, instead of a functional purpose, e.g. a reservoir, for several reasons. The Vasca Votiva is at the top of a terrace/hill (a visible locus for a surrounding area) and not in the center of a village. There are no visible drains or channels linked with it. The sediment laminations in the Vasca Votiva comprise thin laminations from relatively undisturbed continuous decantation. There is no evidence of cleaning out of sediment fill—as might be expected in a regularly used reservoir (or well or canal, etc.)—or of any disturbance. This is in contrast to the situation of a regularly used well or spring or reservoir source [5, 46]. These would not be expected to exhibit such thin, fine, laminae (grading finer upwards from a process of continuous and gentle decantation). Rather, they would be expected to reflect turbulent events and activities linked with physical extraction and the movement of water. For these reasons, it appears that the Vasca Votiva had (primarily or at least in part) another purpose, not functionally related to water harvest or storage, but, given the location, and the impressive set of material culture deposited within it, perhaps linked with other cultural or ideational practices or beliefs and enactments or celebrations of these.

## Conclusions

The dendrochronological analysis of timbers from the Noceto site, indicating likely tree cutting and use dates within the site tree-ring series for the lower and upper tanks, combined with ^14^C wiggle-matching dating, permits us to place the Noceto site specifically at the transition between the BM2 and BM3 phases of the Middle Bronze Age in northern Italy. This transition represents a key event in the development of the Terramare civilization of the region, as it corresponds with a striking and much discussed change in settlement patterns, to a major demographic increase (or change) and marked modifications in land use [5, 11, 43, 45, 47], and more generally to an increase in markers of social complexity. A landscape previously characterized by (an increasing number of) small scattered villages witnessed a transformation with a shift to fewer, much larger, settlements that appear to have acted as central places within a (now) highly exploited territory devoted to relatively intensive plow and irrigation-based agriculture.

The Vasca di Noceto monument, a unique case from archaeological work so far in the region, is striking as a large-scale monument from the BM2–BM3 transition. The construction effort involved, twice within about 12 years, would have required a considerable amount of coordinated work and the existence of directed social-political commitment and focus. It implies some forms of social organization, likely hierarchy, and leadership roles, to enable and articulate collective decision making and activity. However, Terramare settlements are notable for the absence of evidence for visible manifestations of leaders or of an elite [47]. Nonetheless, Terramare settlements, and the associated landscaping with impressive ditches and embankments and irrigation systems, exhibit considerable evidence for the practice of just such collective and cooperative behaviour [47]. Some form of community-based (corporate) entrepreneurship is also proposed to explain the striking evidence for a large-scale intensive textile industry at the near-contemporary Terramare site at Montale [43].

It must be noted as a caveat that is always questionable to impute a ritual association merely because no obvious practical function is evident. Similarly, practical and ritual use of the same space are more than possible (whether at the same or different times). However, the unusual Vasca Votiva tanks, and the absence of any good indication for their use for any regular, let alone practical, activity—versus relatively limited depositions into tanks that were largely left alone with gradual decantation of a sediment fill—all point towards some more reflective (viewing), ideological/ideational, or ritual, role (much though this sphere of human life is inherently often more difficult for archaeology to elucidate [48, 49]). The importance of water and its manipulation to the Terramare world is clear: the Vasca Votiva perhaps embodied some or all aspects of these relationships and meanings as both place and display. If so, then such an apparent ritual (votive) association for the “imposing ritual basin of Noceto” [43] tends to conform with a global pattern where many early monumental constructions appear linked to ritual and ideational projects of various forms (e.g. [50–54]). The combination of place, practice (perhaps with group assembly), belief and (likely some form of) supernatural association seems to enable and permit a social and political focus and set of corporate practices to become possible. In this way such projects are capable of representing and defining both immediate secular aspirations (of a leader(s) or ritually-empowered grouping or corporate grouping) as well as conforming with and fulfilling the long-accepted and generally held sacred values of the wider society. In turn, the very process and act of monumental construction, of place- and memory-making, can reflexively help shape what becomes a new social, political, and economic formation as evident in the BM3 period that comes to mark a major transition in human history in northern Italy.

## Supporting information

S1 File The Noceto tree-ring measurement series employed in this study in Tucson.rwl format. Values reported in 1/100ths of a mm. 999 = end of series. NOC030 = NOC-3, 4 & 18; NOC140 = NOC-14, 19 & 20; NOC060 = NOC-6, 8, 12, 15, 16 & 20; NOC251 = NOC-25; NOC050 = NOC-5 & 17; NOC071 = NOC-7; NOC020 = NOC-2 & 9; NOC101 = NOC-10; NOC231 = NOC-23; NOC271 = NOC-27; NOC221 = NOC-22; NOC261 = NOC-26; and NOC111 = NOC-11.
(DOCX)

S2 File The OxCal runfile for the model shown in Fig 15. (DOCX)

S3 File The OxCal runfile for the model shown in Fig 17A and 17B. (DOCX)
