# Supplementary material for: Dating the Noceto Vasca Votiva, a unique wooden structure of the 15th century BCE, and the timing of a major societal change in the Bronze Age of northern Italy
Source: PLoS One. 2021 Jun 9;16(6):e0251341. doi: 10.1371/journal.pone.0251341 (PMC8189450; doi:10.1371/journal.pone.0251341)
Supplement: S1 File — Values reported in 1/100ths of a mm. 999 = end of series. NOC030 = NOC-3, 4 & 18; NOC140 = NOC-14, 19 & 20; NOC060 = NOC-6, 8, 12, 15, 16 & 20; NOC251 = NOC-25; NOC050 = NOC-5 & 17; NOC071 = NOC-7; NOC020 = NOC-2 & 9; NOC101 = NOC-10; NOC231 = NOC-23; NOC271 = NOC-27; NOC221 = NOC-22; NOC261 = NOC-26; and NOC111 = NOC-11. (DOCX) [file pone.0251341.s001.docx]

**The Noceto tree-ring measurement series employed in this study in Tucson .rwl format. Values reported in 1/100ths of a mm. 999 = end of series. NOC030 = NOC-3, 4 & 18; NOC140 = NOC-14, 19 & 20; NOC060 = NOC-6, 8, 12, 15, 16 & 20; NOC251 = NOC-25; NOC050 = NOC-5 & 17; NOC071 = NOC-7; NOC020 = NOC-2 & 9; NOC101 = NOC-10; NOC231 = NOC-23; NOC271 = NOC-27; NOC221 = NOC-22; NOC261 = NOC-26; and NOC111 = NOC-11.**

NOC030 1001 173 110 109 143 138 124 153 113 102

NOC030 1010 185 173 179 131 102 135 127 90 139 170

NOC030 1020 127 110 97 89 119 119 131 109 159 123

NOC030 1030 70 99 134 145 126 141 129 132 135 125

NOC030 1040 120 95 118 96 76 67 120 122 78 121

NOC030 1050 113 151 147 149 152 123 122 138 129 135

NOC030 1060 143 96 91 78 92 111 98 121 139 165

NOC030 1070 139 91 131 140 162 142 187 152 201 199

NOC030 1080 160 154 148 119 116 124 95 85 120 165

NOC030 1090 130 160 141 123 109 139 162 159 150 135

NOC030 1100 152 152 133 185 149 135 116 87 85 86

NOC030 1110 81 85 109 124 99 131 166 153 146 175

NOC030 1120 149 139 139 116 123 151 129 150 95 127

NOC030 1130 136 136 124 113 124 102 122 122 145 132

NOC030 1140 131 129 138 150 126 120 114 95 87 92

NOC030 1150 86 119 130 138 133 149 142 138 107 116

NOC030 1160 114 110 128 166 123 145 115 100 98 109

NOC030 1170 109 94 74 80 70 58 87 89 82 70

NOC030 1180 68 109 119 102 104 126 159 163 162 121

NOC030 1190 101 118 160 165 143 102 124 174 182 176

NOC030 1200 279 301 143 86 147 140 999

NOC140 982 56 45 68 57 58 54 60 54

NOC140 990 50 41 30 38 45 65 60 62 56 73

NOC140 1000 72 93 94 94 100 100 113 133 105 80

NOC140 1010 82 80 94 88 86 88 87 76 87 92

NOC140 1020 60 70 84 81 101 104 108 59 89 88

NOC140 1030 50 54 64 65 85 88 106 125 111 112

NOC140 1040 103 130 117 118 75 79 97 102 98 129

NOC140 1050 108 141 148 102 83 78 90 82 72 88

NOC140 1060 128 71 89 80 91 119 101 135 169 189

NOC140 1070 178 100 123 149 219 229 229 185 179 186

NOC140 1080 173 177 193 147 165 186 140 171 220 230

NOC140 1090 206 233 213 128 122 131 198 240 213 175

NOC140 1100 142 179 221 262 187 141 158 111 122 187

NOC140 1110 220 187 189 181 136 179 158 158 193 183

NOC140 1120 162 193 213 193 195 235 186 197 116 135

NOC140 1130 195 171 208 187 192 186 173 195 222 195

NOC140 1140 188 181 187 205 206 198 187 168 160 195

NOC140 1150 133 182 184 168 138 145 105 123 104 99

NOC140 1160 100 111 125 165 167 186 162 180 177 241

NOC140 1170 246 218 147 164 187 178 252 223 204 192

NOC140 1180 166 221 204 205 189 220 234 213 180 142

NOC140 1190 139 129 161 149 150 143 151 161 150 167

NOC140 1200 185 149 95 106 123 999

NOC060 1017 83 99 133

NOC060 1020 127 119 107 96 113 90 106 108 161 146

NOC060 1030 95 103 117 149 159 205 207 199 206 197

NOC060 1040 172 183 167 150 118 102 173 163 116 174

NOC060 1050 140 187 227 169 182 192 167 134 129 133

NOC060 1060 174 128 126 97 128 163 142 167 187 165

NOC060 1070 169 97 106 121 136 144 173 159 178 193

NOC060 1080 176 166 181 149 131 125 111 168 193 206

NOC060 1090 172 207 180 130 130 126 158 161 116 104

NOC060 1100 102 131 152 161 155 130 115 90 79 181

NOC060 1110 166 150 151 160 122 138 131 126 140 150

NOC060 1120 137 149 176 191 164 190 177 157 136 202

NOC060 1130 222 192 221 172 151 116 125 158 153 160

NOC060 1140 149 141 164 185 207 196 192 171 179 168

NOC060 1150 130 171 187 153 149 158 151 171 138 144

NOC060 1160 141 156 149 169 174 213 139 150 171 215

NOC060 1170 182 219 155 175 170 160 208 217 187 144

NOC060 1180 147 202 204 229 218 214 214 194 174 130

NOC060 1190 132 120 143 154 142 130 142 123 141 129

NOC060 1200 149 138 128 135 999

NOC251 1104 79 52 58 53 57 155

NOC251 1110 105 70 83 102 114 162 102 91 175 116

NOC251 1120 95 84 80 73 47 74 50 47 41 44

NOC251 1130 60 85 80 60 56 62 104 80 100 103

NOC251 1140 94 114 78 89 53 53 35 32 31 40

NOC251 1150 58 85 99 113 88 70 79 77 48 106

NOC251 1160 118 157 171 176 151 181 161 65 50 111

NOC251 1170 159 190 121 174 168 163 168 171 152 87

NOC251 1180 65 94 118 180 217 231 217 233 194 138

NOC251 1190 129 97 119 164 182 184 157 127 137 999

NOC050 1009 82

NOC050 1010 110 84 92 99 94 94 79 68 79 101

NOC050 1020 82 67 89 86 98 97 94 57 61 74

NOC050 1030 49 44 44 44 60 64 84 99 98 78

NOC050 1040 93 92 92 92 73 67 87 92 82 83

NOC050 1050 66 82 98 73 62 71 72 72 59 92

NOC050 1060 82 57 66 65 77 101 105 127 135 146

NOC050 1070 137 92 119 134 166 184 162 151 140 144

NOC050 1080 146 145 167 144 127 140 109 134 144 199

NOC050 1090 169 181 181 104 88 98 142 169 145 118

NOC050 1100 103 155 163 176 154 150 150 105 101 143

NOC050 1110 138 113 143 128 104 187 140 140 120 141

NOC050 1120 158 176 158 137 157 216 154 163 108 120

NOC050 1130 156 142 199 166 202 163 170 210 170 149

NOC050 1140 157 149 154 153 155 148 169 166 143 175

NOC050 1150 111 146 140 129 122 153 128 177 130 119

NOC050 1160 113 114 119 136 136 169 150 152 152 195

NOC050 1170 201 190 123 146 157 129 183 160 148 151

NOC050 1180 135 179 175 178 175 169 216 164 143 135

NOC050 1190 144 130 174 192 179 134 150 127 139 134

NOC050 1200 149 146 113 999

NOC071 1005 122 133 158 134 101

NOC071 1010 118 126 142 151 134 125 105 95 135 141

NOC071 1020 104 99 95 77 87 85 106 103 124 143

NOC071 1030 96 97 104 153 153 166 191 173 194 168

NOC071 1040 157 137 136 126 96 104 165 167 108 131

NOC071 1050 124 139 144 162 130 158 116 87 81 70

NOC071 1060 72 75 74 57 71 90 79 92 82 91

NOC071 1070 106 70 75 76 98 100 106 115 129 127

NOC071 1080 132 134 123 112 101 110 93 112 129 144

NOC071 1090 120 116 133 105 108 107 135 131 103 102

NOC071 1100 95 114 135 143 145 127 110 85 92 124

NOC071 1110 129 128 127 123 96 128 128 123 135 131

NOC071 1120 131 126 118 144 138 135 114 112 103 123

NOC071 1130 118 110 127 127 147 113 128 131 127 129

NOC071 1140 130 132 122 137 128 146 118 133 128 125

NOC071 1150 108 142 133 127 108 111 105 118 84 99

NOC071 1160 113 107 108 113 120 148 128 106 104 118

NOC071 1170 101 105 94 115 103 89 87 113 101 84

NOC071 1180 84 96 106 92 81 98 128 103 106 84

NOC071 1190 82 66 999

NOC020 1034 140 167 150 155 138 136

NOC020 1040 153 178 187 159 125 78 202 179 119 187

NOC020 1050 127 180 167 212 233 180 136 138 144 112

NOC020 1060 151 135 123 105 139 158 132 204 197 231

NOC020 1070 239 105 208 169 179 184 231 202 222 205

NOC020 1080 179 182 165 162 193 177 121 114 185 261

NOC020 1090 190 214 166 102 97 121 174 195 198 167

NOC020 1100 202 205 160 222 180 203 152 107 132 171

NOC020 1110 158 162 210 174 138 204 188 155 156 169

NOC020 1120 144 155 162 138 129 160 131 136 113 135

NOC020 1130 153 141 156 150 152 135 152 165 163 141

NOC020 1140 148 148 137 161 177 147 151 146 140 137

NOC020 1150 119 148 170 139 120 143 133 177 133 118

NOC020 1160 122 119 110 119 117 138 104 118 113 118

NOC020 1170 143 143 126 139 120 109 155 129 122 111

NOC020 1180 113 150 133 119 124 132 154 135 106 78

NOC020 1190 100 108 95 999

NOC101 1001 122 100 86 119 101 111 175 131 94

NOC101 1010 138 123 155 117 97 118 87 85 105 147

NOC101 1020 140 102 113 85 88 91 106 120 124 122

NOC101 1030 77 102 137 115 111 120 107 105 105 104

NOC101 1040 98 91 117 87 70 56 84 98 67 93

NOC101 1050 91 106 145 151 176 149 125 149 159 126

NOC101 1060 142 106 99 70 86 96 77 98 115 136

NOC101 1070 112 61 137 160 191 166 199 154 208 188

NOC101 1080 122 127 178 139 137 160 124 122 206 267

NOC101 1090 201 237 261 172 147 156 194 177 158 149

NOC101 1100 156 148 142 191 171 154 123 87 73 105

NOC101 1110 87 78 82 87 71 80 95 121 116 148

NOC101 1120 158 142 130 104 118 135 127 133 93 134

NOC101 1130 151 126 114 97 118 114 123 114 122 92

NOC101 1140 94 103 98 82 81 88 83 78 65 85

NOC101 1150 96 153 189 166 176 183 130 161 116 108

NOC101 1160 121 108 121 122 116 123 88 93 109 116

NOC101 1170 92 86 78 82 75 68 90 90 70 77

NOC101 1180 56 76 90 62 72 77 104 83 91 76

NOC101 1190 64 83 112 129 122 111 128 143 151 999

NOC231 1125 83 77 52 17 170

NOC231 1130 218 238 255 250 201 183 164 147 181 191

NOC231 1140 201 176 198 139 152 124 125 83 72 116

NOC231 1150 154 142 158 147 126 125 131 109 121 82

NOC231 1160 69 67 65 70 71 73 65 54 42 92

NOC231 1170 130 134 57 52 66 80 166 95 121 68

NOC231 1180 74 124 130 137 128 177 999

NOC271 1117 177 108 136

NOC271 1120 154 144 108 97 98 137 77 40 58 78

NOC271 1130 48 55 110 102 77 50 47 63 44 41

NOC271 1140 33 46 40 49 37 37 38 33 30 44

NOC271 1150 28 34 38 49 32 50 49 64 36 35

NOC271 1160 55 74 58 56 43 55 67 57 46 73

NOC271 1170 63 52 47 56 81 83 106 157 165 97

NOC271 1180 71 59 101 131 110 129 124 165 148 153

NOC271 1190 157 125 172 209 196 154 196 102 147 152

NOC271 1200 133 147 168 148 164 139 161 163 169 162

NOC271 1210 133 135 158 188 138 184 238 229 250 999

NOC221 939 78

NOC221 940 115 124 155 110 61 104 166 94 52 82

NOC221 950 85 82 89 93 76 80 69 59 96 121

NOC221 960 96 85 89 95 103 65 72 84 53 92

NOC221 970 106 99 68 90 46 74 62 54 68 59

NOC221 980 87 51 43 71 70 65 54 49 43 54

NOC221 990 56 49 39 42 47 72 73 58 62 68

NOC221 1000 87 88 63 42 67 53 43 63 58 47

NOC221 1010 59 50 82 90 75 99 72 60 70 84

NOC221 1020 69 81 95 57 127 102 171 100 168 159

NOC221 1030 63 75 111 143 132 143 140 157 168 150

NOC221 1040 120 120 104 123 87 83 165 146 112 142

NOC221 1050 118 164 173 98 137 129 113 115 142 154

NOC221 1060 224 152 140 118 95 173 144 176 184 186

NOC221 1070 187 115 143 123 168 161 162 160 181 163

NOC221 1080 152 134 139 116 112 128 111 129 149 154

NOC221 1090 114 160 160 118 104 92 125 135 96 83

NOC221 1100 81 117 130 132 128 119 109 79 64 119

NOC221 1110 130 98 125 132 72 131 126 105 99 169

NOC221 1120 114 116 155 161 125 174 140 120 102 112

NOC221 1130 118 106 134 151 148 85 77 96 124 125

NOC221 1140 106 101 118 159 132 139 104 91 85 106

NOC221 1150 107 127 137 121 113 158 126 126 93 100

NOC221 1160 99 163 146 183 155 207 117 145 176 170

NOC221 1170 177 181 138 153 125 104 142 124 114 95

NOC221 1180 85 104 120 123 122 135 159 147 160 109

NOC221 1190 89 70 84 100 110 94 115 89 111 91

NOC221 1200 131 101 104 93 99 74 75 62 67 86

NOC221 1210 78 86 102 89 57 56 89 999

NOC261 1132 196 160 210 88 96 115 148 167

NOC261 1140 122 106 129 160 275 177 132 140 127 116

NOC261 1150 89 113 135 148 114 152 195 118 117 117

NOC261 1160 122 238 83 123 110 132 105 127 110 129

NOC261 1170 136 193 281 194 161 209 294 219 199 140

NOC261 1180 135 244 260 223 245 198 165 131 121 51

NOC261 1190 42 38 46 58 85 81 90 85 100 99

NOC261 1200 124 112 116 50 103 69 78 83 87 128

NOC261 1210 114 129 166 164 147 138 999

NOC111 1022 176 162 137 156 176 227 172 196

NOC111 1030 96 136 234 312 241 293 267 317 322 255

NOC111 1040 222 156 164 136 102 74 159 234 168 163

NOC111 1050 197 244 287 149 228 222 124 124 155 188

NOC111 1060 147 156 135 141 116 181 136 189 182 177

NOC111 1070 162 108 115 149 139 182 175 128 153 164

NOC111 1080 147 199 166 107 127 138 111 98 189 157

NOC111 1090 119 129 132 87 90 84 117 125 84 110

NOC111 1100 96 107 152 149 115 149 150 112 88 211

NOC111 1110 321 211 219 189 102 176 115 96 140 199

NOC111 1120 104 129 127 126 87 153 109 106 52 103

NOC111 1130 99 92 188 179 171 94 94 210 167 164

NOC111 1140 128 141 173 257 196 193 150 129 198 172

NOC111 1150 153 129 178 188 165 189 179 164 186 126

NOC111 1160 190 207 167 205 171 169 135 149 118 239

NOC111 1170 239 307 193 197 202 160 178 179 156 171

NOC111 1180 133 157 162 164 146 200 215 149 137 84

NOC111 1190 86 64 96 89 108 93 101 116 136 129

NOC111 1200 163 999
