# Supplementary material for: Dating the Noceto Vasca Votiva, a unique wooden structure of the 15th century BCE, and the timing of a major societal change in the Bronze Age of northern Italy
Source: PLoS One. 2021 Jun 9;16(6):e0251341. doi: 10.1371/journal.pone.0251341 (PMC8189450; doi:10.1371/journal.pone.0251341)
Supplement: S2 File — (DOCX) [file pone.0251341.s002.docx]

**OxCal runfile for Figure 15 model.** The two data that are discussed as outliers are indicated. This version runs the model minus these outliers. Remove the // comments in each case for the original model, and, for GrM-17406, adjust the preceding Gap statement to 20 years.

Options()

{

Resolution=1;

};

Plot()

{

Outlier_Model("SSimple",N(0,2),0,"s");

D_Sequence ("Noceto")

{

R_Date("NOC-14A GrM-17548 RY995-999@997",3380,25)

{

color="black";

Outlier("SSimple",0.05);

};

Gap(5);

R_Date("NOC-14A GrM-17645 RY1000-1004@1002",3375,25)

{

color="black";

Outlier("SSimple",0.05);

};

Gap(50);

R_Combine ("RY1050-1054= 1052")

{

color="black";

Outlier("SSimple",0.05);

R_Date ("NOC-12-A1 GrM-11242 RY1050-1054@1052",3320,25)

{

Outlier("SSimple",0.05);

};

R_Date ("NOC-14A GrM-13697 RY1050-1054@1052",3274,15)

{

Outlier("SSimple",0.05);

};

};

Gap(10);

R_Date("NOC-14A GrM-13749 RY1060-1064@1062",3332,15)

{

color="black";

Outlier ("SSimple",0.05);

};

Gap(20);

R_Combine ("RY1080-1084= 1082")

{

color="black";

Outlier("SSimple",0.05);

R_Date ("NOC-12-A2 GrM-11243 RY1080-1084@1082",3360,25)

{

Outlier("SSimple",0.05);

};

R_Date ("NOC-14A GrM-13750 RY1080-1084@1082",3332,15)

{

Outlier("SSimple",0.05);

};

};

Gap(20);

R_Date ("NOC-14A GrM-13751 RY1100-1104@1102",3317,15)

{

color="black";

Outlier ("SSimple",0.05);

};

Gap(20);

R_Combine ("RY1120-1124@1122")

{

color="black";

Outlier("SSimple",0.05);

//R_Date ("NOC-12-A3 GrM-11331 RY1120-1124@1122",3227,14)

//{

// Outlier("SSimple",0.05);

//};

//ca.49% outlier

R_Date ("NOC-12A GrM-13696 RY1120-1124@1122",3282,15)

{

Outlier("SSimple",0.05);

};

R_Date ("NOC-14A GrM-13752 RY1120-1124@1122",3260,15)

{

Outlier("SSimple",0.05);

};

R_Date ("NOC-14A GrM-17679 RY1120-1124@1122",3255,25)

{

Outlier("SSimple",0.05);

};

};

Gap(20);

R_Date ("NOC-14A GrM-13754 RY1140-1144@1142",3263,15)

{

color="black";

Outlier ("SSimple",0.05);

};

Gap(40);

//GrM-17406 replaces GrM-11275

//R_Date ("NOC-12-A4 GrM-17406 RY1160-1164@1162",3250,25)

//{

// color="black";

// Outlier ("SSimple",0.05);

//};

//Gap(20);

//ca.12% outlier

R_Date ("NOC-14A GrM-13755 RY1180-1184@1182",3222,15)

{

color="black";

Outlier ("SSimple",0.05);

};

Gap(20);

R_Date ("NOC-14A GrM-17737 RY1200-1204@1202",3160,35)

{

color="black";

Outlier ("SSimple",0.05);

};

};

};
