# Supplementary material for: Dating the Noceto Vasca Votiva, a unique wooden structure of the 15th century BCE, and the timing of a major societal change in the Bronze Age of northern Italy
Source: PLoS One. 2021 Jun 9;16(6):e0251341. doi: 10.1371/journal.pone.0251341 (PMC8189450; doi:10.1371/journal.pone.0251341)
Supplement: S3 File — (DOCX) [file pone.0251341.s003.docx]

1. **OxCal runfile for Figure 17A model.**

Options()

{

Resolution=1;

};

Plot()

{

Outlier_Model("General",T(5),U(0,4),"t");

Outlier_Model("Charcoal",Exp(1,-10,0),U(0,3),"t");

Sequence("Noceto Tanks")

{

Boundary("Start Noceto Lower Tank");

C_Date("RY1206",-1444,4)

{

color="brown";

};

Phase("Lower Tank Use")

{

R_Date("UGAMS-29349 US172a oak gall",3160,25)

{

color="blue";

Outlier ("General",0.05);

};

R_Date("UGAMS-29350 US555 dogwoodberry stone",3080,25)

{

color="blue";

Outlier ("General",0.05);

};

};

Boundary("Transition Lower to Upper Tank");

Phase ("Upper Tank")

{

Sequence("Construction to Use")

{

Phase("Construction")

{

R_Date("Poz-25259 US148 wood",3225,35)

{

color="red";

Outlier ("Charcoal",1);

};

C_Date("RY1218",-1432,4)

{

color="brown";

};

};

Boundary("Transition Construction to Use");

Phase("Use Upper Tank")

{

R_Date("Poz-23426 US5002 bone collagen",3125,35)

{

color="red";

Outlier ("General",0.05);

};

R_Date("Poz-19307 US5003 hazel shell",3085,35)

{

color="red";

Outlier ("General",0.05);

};

R_Date("Poz-19036 US66 hazel shell",3075,35)

{

color="red";

Outlier ("General",0.05);

};

R_Date("Poz-25258 US81 dogwoodberry stone",3115,35)

{

color="red";

Outlier ("General",0.05);

};

Interval("Interval Use Upper Tank");

Date("Date Upper Tank Use");

};

};

};

Boundary("End Noceto Tanks");

};

};

1. **OxCal runfile for Figure 17B model (re-assigning UGAMS-29350).**

Options()

{

Resolution=1;

};

Plot()

{

Outlier_Model("General",T(5),U(0,4),"t");

Outlier_Model("Charcoal",Exp(1,-10,0),U(0,3),"t");

Sequence("Noceto Tanks")

{

Boundary("Start Noceto Lower Tank");

C_Date("RY1206",-1444,4)

{

color="brown";

};

Phase("Lower Tank Use")

{

R_Date("UGAMS-29349 US172a oak gall",3160,25)

{

color="blue";

Outlier ("General",0.05);

};

};

Boundary("Transition Lower to Upper Tank");

Phase ("Upper Tank")

{

Sequence("Construction to Use")

{

Phase("Construction")

{

R_Date("Poz-25259 US148 wood",3225,35)

{

color="red";

Outlier ("Charcoal",1);

};

C_Date("RY1218",-1432,4)

{

color="brown";

};

};

Boundary("Transition Construction to Use");

Phase("Use Upper Tank")

{

R_Date("Poz-23426 US5002 bone collagen",3125,35)

{

color="red";

Outlier ("General",0.05);

};

R_Date("Poz-19307 US5003 hazel shell",3085,35)

{

color="red";

Outlier ("General",0.05);

};

R_Date("Poz-19036 US66 hazel shell",3075,35)

{

color="red";

Outlier ("General",0.05);

};

R_Date("Poz-25258 US81 dogwoodberry stone",3115,35)

{

color="red";

Outlier ("General",0.05);

};

R_Date("UGAMS-29350 US555 dogwoodberry stone re-assigned",3080,25)

{

color="orange";

Outlier ("General",0.05);

};

Interval("Interval Use Upper Tank");

Date("Date Upper Tank Use");

};

};

};

Boundary("End Noceto Tanks");

};

};
